# Primary Cilia Are Lost in Preinvasive and Invasive Prostate Cancer

**DOI:** 10.1371/journal.pone.0068521

**Published:** 2013-07-02

**Authors:** Nadia B. Hassounah, Ray Nagle, Kathylynn Saboda, Denise J. Roe, Bruce L. Dalkin, Kimberly M. McDermott

**Affiliations:** 1 The University of Arizona Cancer Center, University of Arizona, Tucson, Arizona, United States of America; 2 Department of Cellular and Molecular Medicine, University of Arizona, Tucson, Arizona, United States of America; 3 Bio5 Institute, University of Arizona, Tucson, Arizona, United States of America; 4 Department of Urology, University of Washington, Seattle, Washington, United States of America; University of Missouri-Columbia, United States of America

## Abstract

Prostate cancer is the second most commonly diagnosed cancer in men worldwide. Little is known about the role of primary cilia in preinvasive and invasive prostate cancer. However, reduced cilia expression has been observed in human cancers including pancreatic cancer, renal cell carcinoma, breast cancer, cholangiocarcinoma, and melanoma. The aim of this study was to characterize primary cilia expression in preinvasive and invasive human prostate cancer, and to investigate the correlation between primary cilia and the Wnt signaling pathway. Human prostate tissues representative of stages of prostate cancer formation (normal prostate, prostatic intraepithelial neoplasia (PIN), and invasive prostate cancer (including perineural invasion)) were stained for ciliary proteins. The frequency of primary cilia was determined. A decrease in the percentage of ciliated cells in PIN, invasive cancer and perineural invasion lesions was observed when compared to normal. Cilia lengths were also measured to indirectly test functionality. Cilia were shorter in PIN, cancer, and perineural invasion lesions, suggesting dysfunction. Primary cilia have been shown to suppress the Wnt pathway. Increased Wnt signaling has been implicated in prostate cancer. Therefore, we investigated a correlation between loss of primary cilia and increased Wnt signaling in normal prostate and in preinvasive and invasive prostate cancer. To investigate Wnt signaling in our cohort, serial tissue sections were stained for β-catenin as a measure of Wnt signaling. Nuclear β-catenin was analyzed and Wnt signaling was found to be higher in un-ciliated cells in the normal prostate, PIN, a subset of invasive cancers, and perineural invasion. Our results suggest that cilia normally function to suppress the Wnt signaling pathway in epithelial cells and that cilia loss may play a role in increased Wnt signaling in some prostate cancers. These results suggest that cilia are dysfunctional in human prostate cancer, and increase Wnt signaling occurs in a subset of cancers.

## Introduction

**Figure 1:**
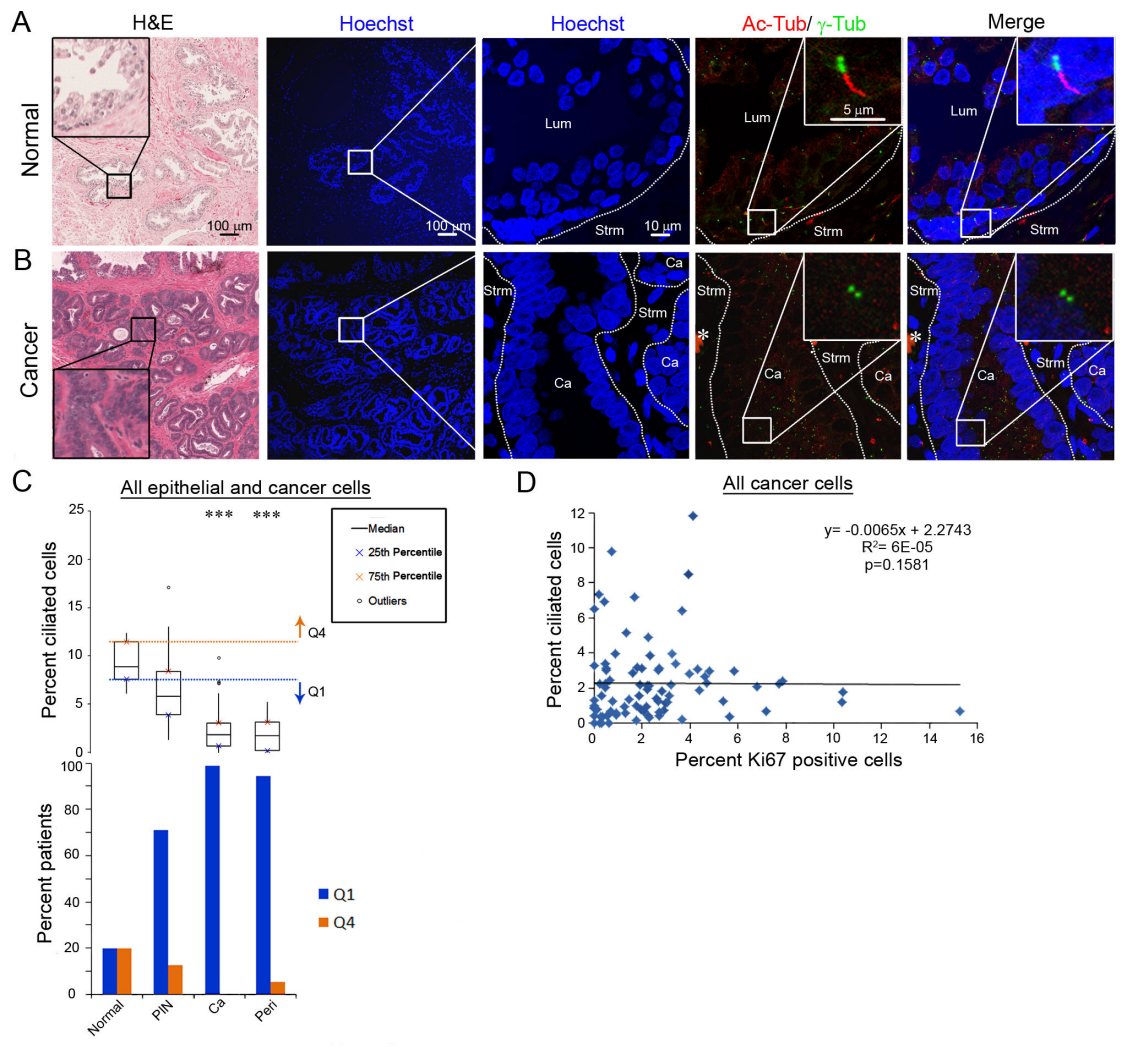
Primary cilia expression is decreased in preinvasive and invasive prostate cancer. Images of (A) normal prostate and (B) invasive prostate cancer. Serially adjacent slides were stained with H&E to visualize tissue morphology or stained fluorescently for nuclei (Hoechst; blue), primary cilia (Ac-Tub; red) and centrosomes (γ-Tub; green). Labeled structures are lumen (Lum), cancer (Ca), and stroma (Strm). Inset shows magnification of (A) a primary cilium on a normal epithelial cell and (B) a centrosome without a cilium on a cancer cell. Asterisk denotes nonspecific staining (C, top). Box plot of the percent of ciliated epithelial and cancer cells per patient for each tissue type: normal, prostatic intraepithelial neoplasia (PIN), cancer (Ca), and perinerual invasion (Peri). Orange line and arrow correspond to Q4 (quartile 4; greater than the 75^th^ percentile for normal tissue) and blue line and arrow correspond to Q1 (quartile 1; less than or equal to the 25^th^ percentile for normal tissue). Statistics were performed using linear regression (*** = p<0.001) (C, bottom). The percent of patients with an abnormally high percent cilia (Q4; orange) or an abnormally low percent cilia (Q1; blue). (D) Percent of Ki67 positive invasive cancer cells and perineural invasion cancer cells per patient (x-axis) versus percent ciliated cancer cells for the same patient (y-axis). Statistical analysis was a non-parametric spearman correlation.

The primary cilium is a microtubule-based organelle that protrudes from the plasma membrane and acts much like an ‘antenna’ to sense extracellular signals. Primary cilia are usually immotile but can sense physical and chemical signals. Cells with primary cilia have only a single cilium extending from the cell surface. At the base of the primary cilium is the basal body (also known as the mother centriole), which is anchored to the plasma membrane. The basal body nucleates the microtubule bundles that extend up the cilium (Figure 1A). Hundreds of proteins have been identified that make up the primary cilium [1]. Many of these proteins localize to the cilium to regulate the sensory or signaling functions of the primary cilium. Cilia act like antennae through sensing extracellular signals and help regulate cell signaling; for example, primary cilia are negative regulators of the Wnt pathway [2,3]. Specifically, primary cilia dampen the Wnt signaling response by compartmentalizing Wnt signaling proteins, such as the positive regulator Jouberin [3]. Cilia have a demonstrated role in developmental biology and human diseases known as ciliopathies (e.g. Joubert syndrome (JBTS), polycystic kidney disease (PKD), Bardet–Biedl syndrome (BBS), and nephronophthisis (NPHP)) [4].

Cilia also play a causal role in tumorigenesis [5,6]. Mouse models demonstrate that in some contexts cilia are required to promote cancer while in other environments loss of cilia increases tumor incidence. Reduced cilia expression has been observed in human cancers including pancreatic cancer, renal cell carcinoma, breast cancer, cholangiocarcinoma, and melanoma [7–11]. These studies support the hypothesis that primary cilia can act as a tumor suppressor organelle in some tissues. Loss of primary cilia was further demonstrated in premalignant pancreatic intraepithelial neoplasia, suggesting that cilia loss may need to occur early to allow for pancreatic cancer formation [10].

The frequency and functionality of primary cilia in preinvasive and invasive human prostate cancer has not been carefully characterized. Prostate cancer is the second most commonly diagnosed cancer and the sixth leading cause of cancer-related deaths in men worldwide. The canonical Wnt signaling pathway has been implicated in human prostate cancer, but the role of this pathway in prostate cancer is not fully understood. The current study is aimed at characterizing primary cilia frequency and function in human prostate cancer and to examine the correlation between primary cilia expression and Wnt signaling. We demonstrate that primary cilia frequency is decreased in all stages of prostate cancer, from early preinvasive lesions to invasive stages. Primary cilia lengths are also decreased in preinvasive and invasive prostate cancer, suggesting loss of function. We further demonstrate that cilia absence correlates with increased nuclear β-catenin localization in normal prostate tissue, and nuclear β-catenin is upregulated in PIN, a subset of prostate cancers, and perineural invasion areas.

## Materials and Methods

### Human tissue specimens

All formalin-fixed paraffin-embedded tissue samples were obtained from the Tissue Acquisition and Cellular/Molecular Analysis Shared Service (TACMASS) at the University of Arizona Cancer Center. Prostatectomy tissue samples were acquired from patients undergoing open radical prostatectomies from 2006 to 2009. Tissue from only one patient was acquired through transurethral resection of the prostate. For the prostate tissue, a total of 32 blocks from 25 cases were chosen based on the presence of areas of interest, which were identified by a pathologist looking at the archived hematoxylin and eosin (H&E)-stained tissue slide for each tissue block. Out of the 25 cases, 23 cases had a cancer located on the tissue block. In seven cases, two tissue blocks were used from the same patient to obtain all the tissue areas of interest. Tissue microarray (TMA) slides were also used from tissue acquired from patients undergoing open radical prostatectomies from 1992 to 2001. Five TMA slides containing ~54 cores (1 mm each) per slide were utilized. Each patient sample was represented four times among the TMA slides with three cores from tumor tissue and one core from normal tissue. From the TMA slides, tissue cores from a total of 53 patients were chosen based on the presence of usable tissue with cancer and/or perineural invasion lesions. Of the TMA tissue from the 53 patients, 1 patient had only perineural invasion lesions. For all the prostate tissue samples described, a single urologist performed the surgeries. Patient and clinical data was also obtained for most patients, including age at surgery, pathological tumor stage, pre-operative free prostate specific antigen (PSA) levels, biochemical recurrence, time to biochemical recurrence, capsular penetration, and largest tumor volume. Written consent was given by the patients for their information to be used for research. Biochemical recurrence was defined as free serum PSA >0.1 ng/ml for two consecutive measurements. The range of the length of time between surgery and last follow-up is 10 months to 16 years. Pathologically normal, cancer-free prostate tissues from 10 patients were used as controls and were obtained from patients with bladder cancer who underwent cystoprostatectomies.

Serial tissue sections were cut for each patient sample. The first serial section was stained for H&E and the entire tissue section was scanned with a 20x objective using the automated DMetrix slide scanner (DMetrix, Inc.). Digital images were annotated using the Eyepiece software (DMetrix, Inc.) by a certified pathologist for each of the areas of interest per tissue slide. For the whole cohort, the areas of interest used were as follows: pathologically normal prostate from bladder cancer patients (n=10), pathologically normal prostate adjacent to cancer (n=16), benign prostatic hyperplasia (BPH; n=8), prostatic intraepithelial neoplasia (PIN; n=24), low-grade PIN (LG PIN; n=13) and high-grade PIN (HG PIN; n=18), low-grade (LG; Gleason sum score =6; n=35) and high-grade cancers (HG; Gleason sum score >6; n=40), and perineural invasion lesions (n=18). All of the tissue types were taken from the radical prostatectomies, except for the pathologically normal prostate tissue from bladder cancer patients (these were from cystoprostatectomies). Seven patients had both LG (increased nuclear-to-cytoplasmic ratio, and increased nuclear size) and HG (presence of prominent nucleoli, increased nuclear-to-cytoplasmic ratio, and increased nuclear size) PIN. Gleason sum score is a grading method used to assess the degree of differentiation [12].

### Immunofluorescence

The tissue slide serial to the H&E-stained slide was used for co-staining of cilia, centrosomes and cytokeratin 5 (CK5). Paraffin-embedded tissue slides were deparaffinized in a dry incubator at 65°C for 15 minutes and hydrated by washing with Xylene (2 x 10 min), 100% Isopropanol (2 x 10 min), 70% Isopropanol (2 x 10 min), 50% Isopropanol (2 x 10 min), and ultrapure water (2 x 10 min). All washes were at room temperature. Antigen retrieval with a 1mM EDTA unmasking solution was performed using a 2100 Retriever (Electron Microscopy Sciences) according to manufacturer’s instructions. Tissue slides were placed in Shandon Coverplates (Thermo Scientific, Cat# 72-110-017) and then into Sequenza Slide Racks (Thermo Scientific, Cat# NC0263065). Tissue slides were blocked with ChemMate Antibody Dilution Buffer (Ventana Medical Systems, Inc., Cat#ADB250) with goat serum (5%) (Invitrogen Corporation, Cat# 16210-064) for 45 minutes at room temperature. Primary and secondary antibodies were diluted in the ChemMate Antibody Dilution Buffer at 1:1000. Primary antibodies were used against acetylated tubulin (mouse monocloncal IgG_2B_, Sigma, Cat# T7451, clone 6-11B-1), γ-tubulin (mouse monoclonal IgG1, Sigma, Cat# T5326, clone GTU-88), and CK5 (rabbit polyclonal, Abcam, Cat# ab24647), and incubated on the tissue overnight at 4°C. Five slides were stained with another primary antibody against CK5 (ready to use mouse monoclonal IgG1, Leica Microsystems, Cat# CK5-R-7-CE) to verify the specificity of the CK5 antibody used. One prostate tissue slide with normal and cancerous areas were also stained with a primary antibody against Arl13b (mouse monoclonal IgG_2a_, UC, Davis/NIH NeuroMab Facility, clone N295B/66) to verify the marking of cilia with the antibody against acetylated tubulin. The slides were then washed with PBS for 10 minutes (3 x 10 min). The secondary antibodies used were tetramethylrhodamine isothiocyanate (TRITC)-labeled goat anti-mouse-IgG_2B_ (Southern Biotech, Cat# 1090-03), Alexa 633-labeled goat anti-mouse-IgG1 (Invitrogen, Cat# A21126), Alexa 546-labeled goat anti-mouse-IgG_2a_ (Invitrogen, Cat#A21133) and fluorescein isothiocyanate (FITC)-labeled goat anti-rabbit-IgG (Southern Biotech, Cat# 4052-02). Secondary antibodies were incubated on the tissue for 45 minutes at room temperature. Slides were washed with PBS for 10 minutes (3 x 10 min). Hoechst 33342 (Cat#H3570, Invitrogen) was used as a counterstain at 1:1000 and incubated on slides for 10 minutes, followed by washing with PBS for 5 minutes (2 x 10 min). Slides were mounted with 1.5 coverslips (0.16-0.19 mm thickness) (Fisher Scientific, Cat# 12-544B) using Prolong Gold Antifade mounting media (Cat# P36934, Invitrogen).

### Confocal imaging

The tissue slide that was serially adjacent to the digitally scanned H&E slide was fluorescently stained for cilia, centrosomes, CK5 and Hoechst. The Leica TCS SP5 II laser scanning confocal microscope (Leica Microsystems) was used to image the fluorescently-stained slides. Areas of interest were found using a low-power magnification objective (10x, 0.4 PI Apo) to visualize Hoechst counterstain on the fluorescently-stained slide and by referencing the annotated serially adjacent H&E that was digitally scanned. This allowed us to find the exact same area of interest that had been annotated by the pathologist on the H&E. Once the area of interest was found, Z stacks were then acquired with the violet-laser diode at 405 nm to detect Hoechst staining at a total thickness of 2 ± 0.5 µm, with a Z-step taken every 1 µm. Cilia were then imaged within these areas of interest using a 63x objective (1.4 NA PL Apo) with the helium neon lasers (543 nm and 633 nm), CK5 was imaged with the argon laser (488 nm), and the violet-laser diode (405 nm) was used to detect Hoechst staining. Z-stacks were acquired at a total thickness of 5.0 ± 0.5 µm, with a Z-step taken every 0.34 µm (image resolution 2048x2048 pixels). We acquired a range of 1-6 images per location using the 63x objective per tissue type per patient and this varied depending on the size of the location. Z images were processed post-acquisition to maximum projections using the Leica LAS AF software for image analysis.

### Confocal image analysis

Cilia frequency and cilia lengths were obtained for each cell type using the Leica LAS AF software. Cilia were only scored when both ciliary axoneme and centrosome were visible together. For each cell type [CK5 positive (CK5+) epithelial/cancer cells, CK5 negative (CK5-) epithelial/cancer cells, and CK5-stromal cells], nuclei were counted using the count tool, and cilia lengths were measured using the scale bar tool. The number of cilia per cell type was divided by total nuclei per cell type to obtain a percentage of ciliated cells. At least 171 nuclei were counted per tissue type per patient (max=2770, median= 782). Median cilia lengths per patient were determined and used in data and statistical analyses. Boxplots illustrate the data, where the 75^th^ and 25^th^ percentiles are marked by the upper and lower box limits, respectively. The black line within the box denotes the median. Outliers are defined as either < 25^th^ percentile -1.5X interquartile range, or >75^th^ percentile + 1.5X interquartile range (open circles). Extreme outliers were defined as either < 25^th^ percentile -3X interquartile range, or >75^th^ percentile + 3X interquartile range (sold circles). We utilized data obtained about percent cilia and cilia length found in normal prostate to establish a cutoff to provide a relative comparison point to cilia found in PIN and cancer samples. The upper and lower 25^th^ percentiles were chosen as our relative normal cutoffs and this was kept consistent throughout our analysis. From the boxplots, the percent of patients with an abnormal percent of ciliated cells or abnormal median cilia length were calculated. These abnormal measurements were defined by the 75^th^ and 25^th^ percentiles of normal. Values were considered abnormally high if they fell above the 75^th^ percentile of normal and abnormally low if they fell at or below the 25^th^ percentile of normal. Nothing is known about precise lengths of cilia needed for normal function. Therefore, our upper and lower 25^th^ percentile cutoff only provide a frame of reference for comparison to normal.

### Immunohistochemistry

Formalin-fixed paraffin-embedded tissue slides were stained with a similar protocol used for immunofluorescently stained slides described above. Antigen retrieval was performed as described but with the Vector Antigen Unmasking Solution (1:106) (Vector Laboratories, Cat# H-3300), followed by quenching of endogenous peroxidase activity at room temperature for 20 minutes using hydrogen peroxide in methanol (0.3%). Slides were then washed with PBS (4 x 10 min), placed into Shandon Coverplates (Thermo Scientific, Cat# 72-110-017) and then into Sequenza Slide Racks (Thermo Scientific, Cat# NC0263065). Slides were then blocked with 2.5% normal horse serum blocking buffer (Vector Laboratories, Cat# S-2012) for 20 minutes, and then with ChemMate Antibody Dilution Buffer (Ventana Medical Systems, Inc., Cat# ADB250) with goat serum (5%) (Invitrogen Corporation, Cat# 16210-064) for 45 minutes. All washes and blocking steps were at room temperature. Primary antibodies were diluted in the ChemMate Antibody Dilution Buffer. The following primary antibodies were used: β-catenin primary antibody (1:400, mouse monoclonal IgG1, BD Transduction Laboratories, Cat#610154) and Ki67 (1:100, mouse monoclonal IgG1, Dako, Cat#M7240, clone MIB-1). Colon adenocarcinoma tissue slides were used as a positive control for β-catenin and Ki67 staining. Tissue slides with secondary antibody only were used as a negative control. Primary antibodies were incubated on tissues overnight at 4°C. Slides were then washed in PBS (3 x 10 min). A universal anti-rabbit, anti-mouse secondary antibody conjugated to peroxidase (ImmPress Universal Reagent, Vector Laboratories, Cat# MP-7500) was incubated on the tissue for 30 minutes, followed by a wash in PBS for 5 minutes. 3-amino-9-ethylcarbazole (AEC) with high sensitivity substrate chromogen (Dako, Cat# K3461) was use as the peroxidase substrate for β-catenin and Ki67 staining. AEC was incubated on slides for 3 minutes for β-catenin staining and 7 minutes for Ki67 staining. Tissue slides were rinsed with distilled water for 5 minutes. Hematoxylin 1 (Thermo Scientific, Cat# 7221) was used to counterstain the tissue slides. Hematoxylin 1 was diluted 1:3 and incubated on tissue slides for 15 seconds and then rinsed in tap water until water ran clear. Faramount Aqueous Mounting Media (Dako, Cat# S3025) was used for mounting slides using 1.5 coverslips (0.16-0.19 mm thickness) (Fisher Scientific, Cat# 12-544B).

### Immunohistochemistry analysis

Whole slides stained for β-catenin were scanned using an automated Dmetrix scanner at 20x magnification (DMetrix Inc.). Slides stained for Ki67 were scanned using a 20x objective with the BioImagene scanner (Ventana Medical Systems). The same locations used for primary cilia analysis were found by referencing the annotated H&E slide. These areas of interest were exported as TIFF files from the Dmetrix scan files, and as JPEG files from the BioImagene scan files, and uploaded into Definiens Tissue Studio 3.0 Software (http://www.tissuestudio.com). Tissue Studio 3.0 Software was tested for absolute agreement with manual hand counts performed by two separate investigators. Images from six normal prostate and six prostate cancer locations were blindly scored for nuclear staining of Gli1, where each nucleus was scored as either positive or negative. For each image, Tissue Studio 3.0 was used in conjunction to quantify the number of positive and negative cells/nuclei. A statistical test that is used to measure the consistency and absolute agreement of measurements made by different observers (intraclass correlation) was applied to the data obtained for the six normal and cancerous prostate tissues. The intraclass correlation coefficient was determined as 0.7, using SPSS 19 (Statistical Package for the Social Sciences; IBM Corporation), which is considered strong agreement.

For Ki67 analysis, some patient tissue could not be used for analysis due to too many serial sections missing between the Ki67-stained slide and the H&E slide, making it impossible to locate the exact area used for primary cilia analysis. The number of patients used for cancer was 72, and the number of patients used for perineural invasion was 15. For the Ki67 analysis with Definiens Tissue Studio, a modified Nuclei (Positive/Negative) solution was used. The epithelial/cancer and stromal compartments of cancer and perineural invasion areas were separately analyzed using the Manual ROI Selection (Select Segments) segmentation tool, with a segmentation of 8. The hematoxylin and immunohistological (IHC) threshold were set at 0.12 arbitrary units (a.u.) and 0.03 a.u., respectively. The IHC threshold was determined by identifying the lightest positively-stained nucleus in the sample set and using this value as the cutoff for positivity. A Nucleus Morphology and Filter step was used to exclude objects mistakenly identified as nuclei. From the exported results, positive indices were computed per tissue type per patient.

Some patient tissue could not be used for β-catenin analysis due to inadequacy of serial sections, or too many serial sections missing between the β-catenin stained slide and the H&E slide, making it impossible to find the exact location. For normal, the number of locations utilized was 26, from 10 patients. For PIN, the number of locations was 19, from 13 patients. For cancer, 154 locations were used, from 64 patients. For perineural invasion lesions, the number of locations used was 23, from 11 patients. For the β-catenin analysis with Definiens Tissue Studio, the percentage and intensity of nuclear staining in epithelial and cancerous tissue was acquired. Stroma was excluded from the analysis since β-catenin is mostly expressed in epithelial cells. For the analysis, a modified Nuclei, Membrane and Cells solution was used. The magnification and resolution was set to 20x and 0.5 µm/pixel, respectively. A Manual ROI Selection (Draw polygons) was used for normal, PIN, cancer, and perineural invasion areas to separately analyze the epithelial compartment. In normal, basal cells, which were identified by position, morphology and according to the CK5-stained serially adjacent tissue section, were selected separately from the luminal cells for analysis. In PIN, cancer, and perineural invasion lesions, the whole epithelial/cancer compartment was selected for analysis. After regions of interest were selected, separate nuclei and cells were identified using manually set parameters and thresholds, followed by classification of nuclei based on manually set thresholds. To identify nuclei and cells, “membrane” was chosen for the IHC marker. For nucleus detection, hematoxylin and IHC thresholds were set at 0.055 arbitrary unit (a.u.) and 1.02 a.u., respectively. For detection of membranes and cells, the IHC threshold was set to 0 a.u., and the membrane thickness to 1 pixel. Nuclei classification thresholds were set to classify a nucleus as having either no, low, medium or high immunohistochemical staining. These thresholds were chosen individually based on slides from a control prostate tissue sample used in every staining run, by identifying the darkest and lightest nuclear staining. The lightest staining was used to set the no/low staining threshold, the highest staining was used for the medium/high threshold, and the value in between those two thresholds were used for the low/medium threshold. The nuclear thresholds for no/low staining ranged from 0.3–0.4 a.u., low/medium staining ranged from 0.45–0.55 a.u., and medium/high staining ranged from 0.6–0.7 a.u.

The histological score was calculated from the following formula: 3X (% nuclei stained high) + 2X (% nuclei stained medium) + 1X (% nuclei stained low). The histological score was determined for each separate location which had been used for cilia analysis, and this was plotted versus percent cilia. It should be noted that there are multiple locations per patient, and this was taken into account during statistical analysis.

### Statistical analysis

Percent ciliated cells and cilia lengths were measured for individual cells by participant and cell type: CK5+, CK5-, and stromal cells. Scatter plots of percent ciliated cells and cilia lengths revealed highly skewed distributions. As a result, medians are reported in tables of summary statistics. Statistical significance was assessed using a linear regression model that clustered on cases in order to control for multiple observations per participant. The dependent variables were log transformed to make them more normal. Differences between diagnostic categories were obtained by fitting indicator variables for each diagnosis. Linear trends in severity of diagnosis was tested by fitting diagnosis as a continuous variable. The correlation between percent cilia and Ki67 score of cancer was performed using a non-parametric Spearman correlation. Associations between percent cilia and patient data were quantified using linear regression. Analysis for differences in nuclear β-catenin was performed using correlation, linear regression, and two-by-two tables. All analyses were performed in STATA (StataCorp). A p-value of less than 0.05 was considered statistically significant. The statistical analyses do not control for multiple comparisons.

## Results

### Primary cilia expression is decreased in preinvasive and invasive prostate cancer

To characterize the frequency of primary cilia in different severities of human prostate cancer, primary cilia presence was determined for the different tissue types: cancer-free normal tissue, prostatic intraepithelial neoplasia (PIN), cancer and perineural invasion lesions (see Table 1 for details). The first serial section was stained with H&E to identify areas of normal, PIN, cancer and perineural invasion lesions. The H&E slide was used as a reference to find the tissue types of interest on the adjacent serial section, which was stained for acetylated tubulin (Ac-Tub) to visualize primary cilia and γ-tubulin (γ-Tub) to identify associated centrosomes (Figure 1A and 1B). Primary cilia frequency and length were quantified. A total of 90,427 nuclei were counted with an average of 20,782 nuclei counted per tissue type and an average range of 248-1603 nuclei per patient (Table S1A in Table S1).

**Table 1:** Summary of patient and clinical data.

	n (%)
**Age at diagnosis (years)**	**n=76**
<60	20 (26.3)
60-64	11 (14.5)
65-69	26 (34.2)
70-74	16 (21.1)
>74	3 (3.9)
Median=65 Mean=64±7 Range=46-77	
**Gleason sum score**	**n=75**
6	35 (46.7)
7-10	40 (53.3)
PIN grade*	**n=24**
Low grade	13 (54.2)
High grade	18 (75)
**Pathological stage**	**n=77**
pT1-pT2	25 (32.5)
pT3	52 (67.5)
**Volume of largest tumor (cc)**	**n=25**
<3.0	8 (32)
3.0-6.0	9 (36)
6.1-9.0	4 (16)
9.1-12	2 (8)
>12	2 (8)
Median=4 Mean=6.3±8.3 Range=0.13-42	
**Pre-operative free serum PSA (ng/ml)**	**n=58**
<3.0	3 (5.2)
3.0-6.0	24 (41.4)
6.1-9.0	15 (25.9)
9.1-12	4 (6.9)
>12	12 (20.7)
Median=6.4 Mean=11.2±20.4 Range=1.8-154	
Biochemical recurrence**	**n=78**
Yes	44 (56.4)
No	34 (43.6)

* Seven patient samples have both low grade and high grade PIN (prostatic intraepithelial neoplasia).

** Biochemical recurrence defined as free serum PSA >0.1 ng/ml for two consecutive measurements. Time from surgery to last recorded PSA measurements for patients without biochemical recurrence ranges from 12.5 months to 15.2 years, and for patients with recurrence the time from surgery to biochemical recurrence ranges from 1.3 months to 13 1 years.

Loss of primary cilia was seen in prostatic preinvasive samples (PIN; low-grade (LG) and high-grade (HG) combined). The median percentage of ciliated epithelial cells in PIN (median=5.7%) decreased 36% compared to normal epithelial cells (median=8.9%; p=0.24; Figure 1C, top and Table S1A in Table S1). While this is not a statistically significant difference, 70.8% of the PIN samples had an abnormally low (falling at or below the 25^th^ percentile of normal, Q1) percentage of ciliated epithelial cells (Figure 1C, bottom and Table S1B in Table S1). There was also an overall significant linear trend to decreasing percent cilia with increasing severity of prostate cancer (p<0.0001; Table S1A in Table S1). Although this model assumes increasing severity from normal, PIN, invasive cancer, and then to perineural invasion, we recognize that not all cancers develop in this sequence. It should be noted that separating the PIN samples into LG and HG groups did not reveal a statistically significant difference in percentage of ciliated cells throughout the study, so LG and HG PIN were not separated for further percent cilia analyses.

Primary cancer and perineural invasion lesions had a statistically significant loss of primary cilia. The median percentage of ciliated prostate cancer cells (median=1.9%; 78.7% decrease; p <0.0001) and perineural invasion lesions (median=1.7%; 80.9% decrease; p <0.0001) were significantly decreased compared to normal epithelial cells (median=8.9%; Figure 1B, 1C, top and Table S1A in Table S1). Also, 98.7% of cancer samples and 94.4% of perineural cancer samples had an abnormally low percentage of ciliated cancer cells compared to normal samples (Figure 1C, bottom and Table S1B in Table S1). As with PIN, separating the cancer samples into LG (Gleason sum score =6) and HG (Gleason sum score >6) groups did not reveal a statistically significant difference in percentage of ciliated cancer cells throughout the study, so LG and HG cancers were not separated for further percent cilia analyses. Taken together, these results demonstrate that loss of primary cilia is a common event in early preinvasive and invasive prostate cancer.

To validate the use of Ac-Tub staining as a marker of cilia we co-stained normal and cancerous prostate tissue with another primary cilia-specific antibody recognizing Arl13b (Figure S1). Ac-Tub and Arl13b always colocalized in cilia of normal and cancerous prostate, demonstrating that lack of detection of cilia in cancer is due to loss of cilia and not loss of acetylation of tubulin.

Primary cilia are expressed on cells in G_0_/early G_1_ of the cell cycle and resorption of primary cilia occurs when the cell enters into the cell cycle to proliferate [13]. To investigate if the loss of primary cilia in cancer cells is due to a high proliferative index we stained the prostate cancer tissue samples with an antibody that recognizes Ki67, a protein expressed in all phases of the cell cycle except G_0_ [14]. We found that the majority of cancers samples (75% of samples) had <3.2% Ki67 positive cells (Figure 1D). This low percentage of Ki67-positive cells therefore does not account for the significant loss of cilia observed in these prostate cancer cells. Furthermore, no statistical correlation was observed between low percent cilia and high percent Ki67 in prostate cancer samples (p=0.16). These results demonstrate that loss of cilia in prostate cancer is not due to increased proliferation.

### Primary cilia frequency is decreased on both CK5 positive and CK5 negative cancer cells

**Figure 2:**
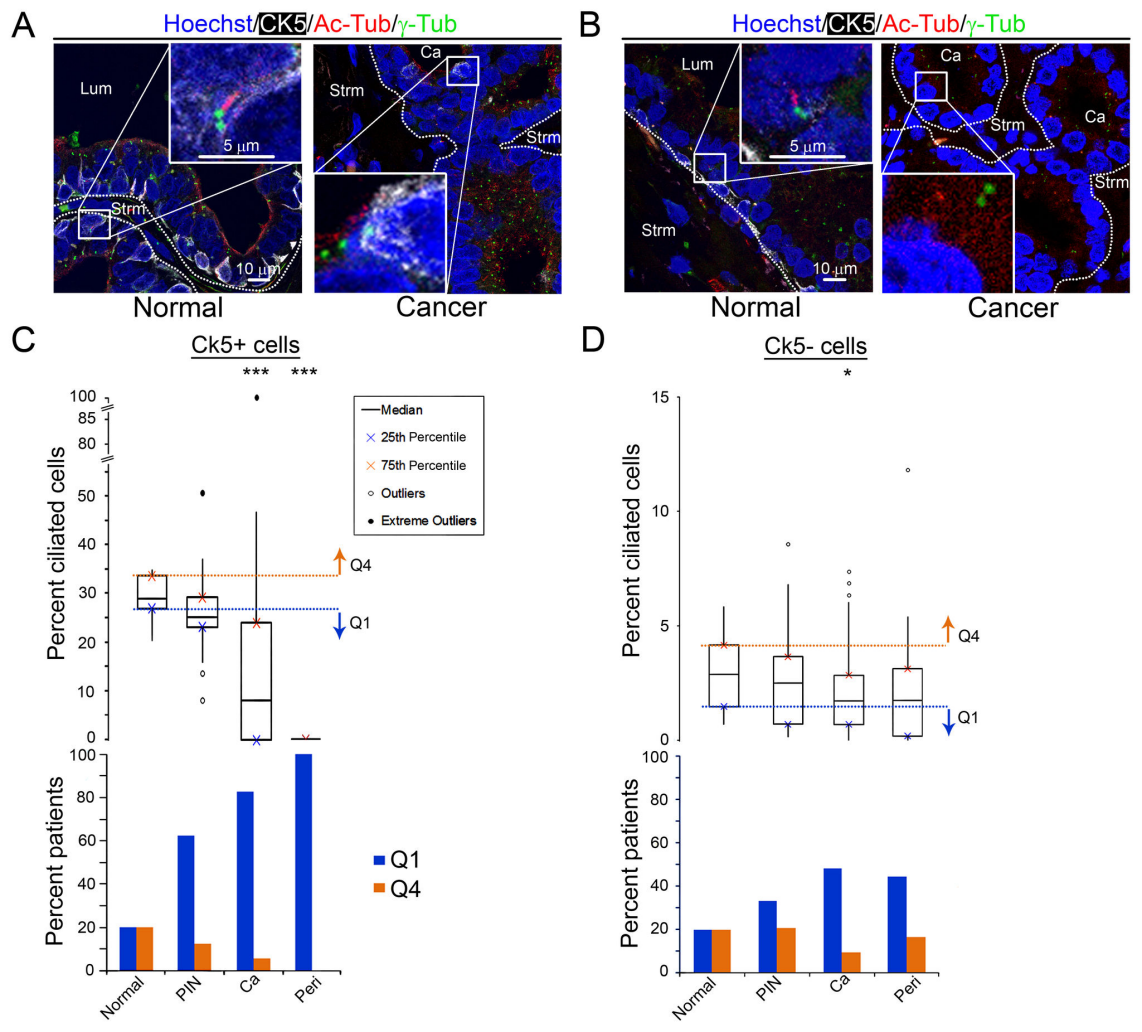
Primary cilia frequency is decreased on both CK5+ and CK5- cancer cells. Images of normal prostate and invasive prostate cancer. Nuclei (Hoechst; blue), cytokeratin 5 (CK5; white), primary cilia (Ac-Tub; red) and centrosomes (γ-Tub; green) are labeled. (A) CK5 marks the basal cell population in normal tissue. (A, left) A ciliated CK5+ normal cell and (A, right) an unciliated CK5+ cancer cell are shown at higher magnification. (B, left) A ciliated CK5- normal cell, and (B, right) an unciliated CK5- cancer cell. (C, top) Boxplots of the percent ciliated CK5+ and (D, top) CK5- epithelial and cancer cells per patient for each tissue type: normal, prostatic intraepithelial neoplasia (PIN), cancer (Ca), and perineural invasion (Peri). Q4, Q1 are as in Fig. 1. Statistics were performed using linear regression (*=p<0.05, ***= p<0.001). (C, bottom) Percent of CK5+ and (D, bottom) CK5- patients with an abnormally high percent cilia (Q4; orange), or an abnormally low percent cilia (Q1; blue).

The normal prostate has a bilayered epithelium of basal cells and secretory luminal epithelial cells [15]. To investigate differences in primary cilia expression on basal versus luminal epithelial cells, we co-stained the prostate tissue cohort for cilia together with an antibody that recognizes cytokeratin 5 (CK5), a basal cell marker. To verify that the CK5 antibody used was specific, we compared it to another commonly used mouse monoclonal antibody (Leica Micosystems, Inc.), and found it to be specific for basal cells (Figure S2E). Tissues were co-stained with antibodies that recognize primary cilia (Ac-Tub and γ-Tub). CK5 positive (CK5+) basal cells were present in all normal tissue with 24.9% of total normal epithelial cells being CK5+ (Figure S2C). All PIN structures also had CK5+ basal cells, with 15.2% of all cells being CK5+. CK5+ expression is generally thought to be lost in prostate cancer, however, like others, we observed rare CK5+ cells in invasive cancers (Figures 2A and S2A) [16–18]. The CK5+ cancer cells were also typically sporadic and not located adjacent to one another. In addition, the CK5+ cancer cells did not have a normal basal morphology (Figure 2 and Figure S2). At least one CK5+ cancer cell was observed in 46.7% of prostate cancers and 33.3% of perineural invasion lesions (Figure S2B). Of all cancer cells and all perineural cancer cells analyzed, 1.6% and 1.3% respectively were CK5+ (Figure S2C).

We wanted to determine if the frequency of primary cilia changes on both the CK5+ and CK5-cells in normal prostate as well as preinvasive and invasive prostate cancer. Primary cilia were enriched 9.9-fold on normal CK5+ cells (median=28.8%) compared to normal CK5-cells (median=2.9%; Table S2A in Table S2 and Figure 2C, 2D, top). The frequency of primary cilia was reduced by 12.5% (median=25.2%; p=0.14) on CK5+ cells associated with PIN. The frequency of primary cilia also decreased 13.8% on CK5-cells (median=2.5%; p=0.4) in PIN. Although this decrease in percent cilia on CK5+ and CK5-of PIN was not statistically significant, 62.5% of patients had an abnormally low percent of ciliated CK5+ cells and 33.3% of patients had an abnormally low percent of ciliated CK5-cells (Figure 2C, 2D, bottom and Table S2B in Table S2). There was also a significant linear trend of decreasing percent cilia with increasing severity of prostate cancer in both CK5+ cells (p<0.0001) and CK5-cells (p=0.049). Overall, the data is consistent with the loss of cilia occurring early in a subset of CK5+ and CK5-prostate cancer cells.

We also quantitated the percent ciliated CK5+ and CK5-cells associated with invasive prostate cancers. There was a statistically significant decrease in the percentage of primary cilia on CK5+ (median=8.1%; 72% decrease; p<0.0001) and on CK5- (median=1.7%; 41% decrease; p=0.02) cancer cells compared to normal (Figure 2 and Table S2A in Table S2). Perineural invasion lesions also showed a drastic loss of cilia on CK5+ cancer cells (median=0%; p<0.0001) and a 41% decrease of cilia on CK5- (median=1.7%; p=0.12) cancer cells compared to normal. The decrease in medians of percent cilia on CK5-perineural invasion cancer cells was the same as for the CK5-invasive cells. In addition, 44.4% of the perineural invasion cases had an abnormally low percent of ciliated cells, which was similar to that seen for the invasive cases (48%; Figure 2D, bottom, Table S2B in Table S2). That this reduction did not reach significance (p=0.12) is likely due to the fewer numbers of perineural invasion cancer (n=18) versus the number of invasive cancers (n=75). The overall linear trend for decreasing percent cilia with increasing severity of prostate cancer was significant (p=0.049). Together, these data suggest that primary cilia are lost on both CK5+ and CK5-invasive prostate and perineural invasion-associated cancer cells.

### Primary cilia expression does not change on stromal cells surrounding cancer

**Figure 3:**
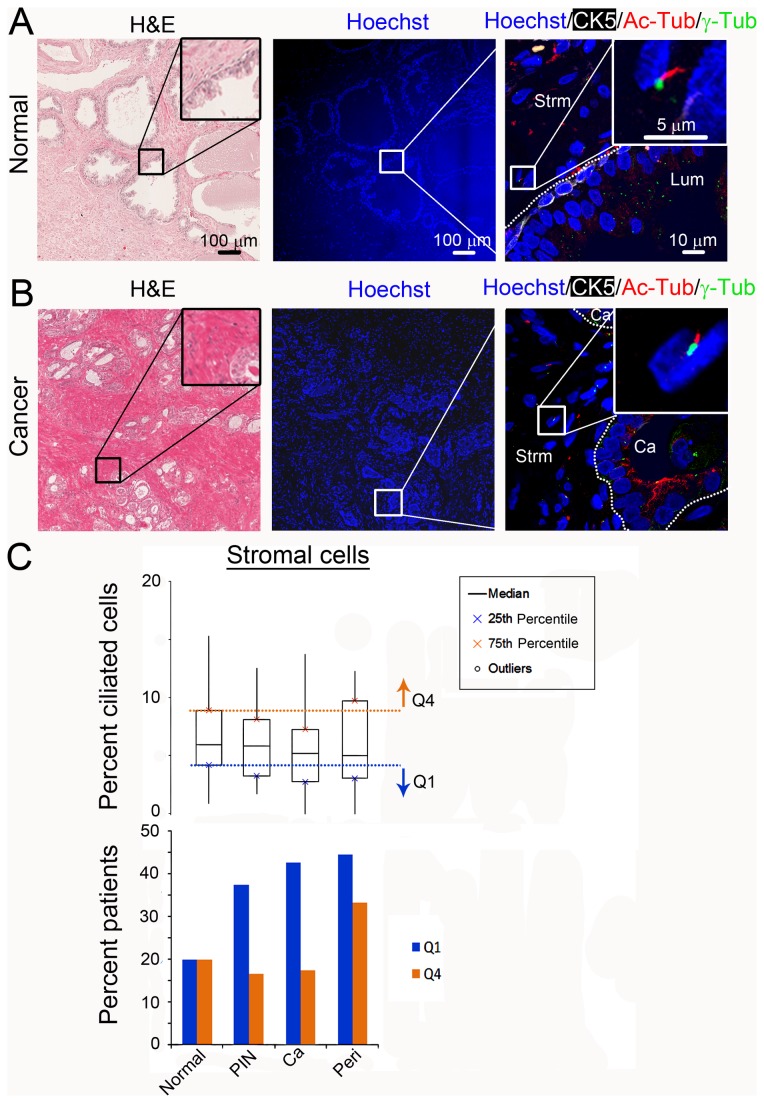
Frequency of cilia does not change on stromal cells surrounding cancer. Images of (A) normal prostate and (B) prostate cancer show nuclei (Hoechst; blue), cytokeratin 5 (CK5; white), primary cilia (Ac-Tub; red) and centrosomes (γ-Tub; green). Inset shows magnification of primary cilia on stromal cells associated with (A) normal tissue and (B) cancerous tissue (C, top). Boxplot describe the percent ciliated stromal cells per patient for each tissue type: normal, prostatic intraepithelial neoplasia (PIN), cancer (Ca), and perineural invasion (Peri). Q4, Q1 are as in Figure 1 (C, bottom). The percent of patients with an abnormally high percent cilia (Q4; orange) or an abnormally low percent cilia (Q1; blue).

The microenvironment of a cancer can contribute to its progression and metastasis [19]. Therefore, we investigated primary cilia changes on stromal cells (identified based on location, morphology, and lack of CK5 staining) surrounding preinvasive and malignant prostate cancer (Tables S3A and S3B). A total of 38,689 nuclei were counted, with an average of 8,650 nuclei per tissue type and an average range of 67-663 nuclei per patient (Table S3A in Table S3). The frequency of primary cilia on stromal cells associated with PIN (median=5.8%; p=0.71), invasive cancer (median=5.2%; p=0.21) and perineural invasion (median=5%; p=0.6) did not change compared to normal (median=5.9%; Figure 3C, top). No significant overall linear trend of decreasing ciliated stromal cells with increasing severity of prostate cancer was observed (p=0.32; Table S3A in Table S3). It should be noted that the diverse types of stromal cells (i.e. fibroblasts, lymphocytes, etc.) were not differentiated, and this may influence the overall percent cilia observed in the stroma. Collectively, these data suggest that primary cilia are lost on preinvasive and invasive cancerous cells, but not on the surrounding stromal cells.

### Ciliated prostate cancer cells as well as stromal cells surrounding cancers have shortened cilia lengths

To determine the potential functionality of cilia that are not lost in prostate cancer cells we measured their lengths (Table S4A in Table S4). Abnormally short cilia have been shown to correlate with loss of function in signal transduction pathways such as Hedgehog signaling [20]. This suggests that abnormal cilia lengths are a convenient indirect measure of the functional state of the cilia. A total of 5,941 cilia were measured on all epithelial and cancer cells, with an average of 730 cilia per tissue type (Table S4A in Table S4). A median cilia length was calculated per patient.

**Figure 4:**
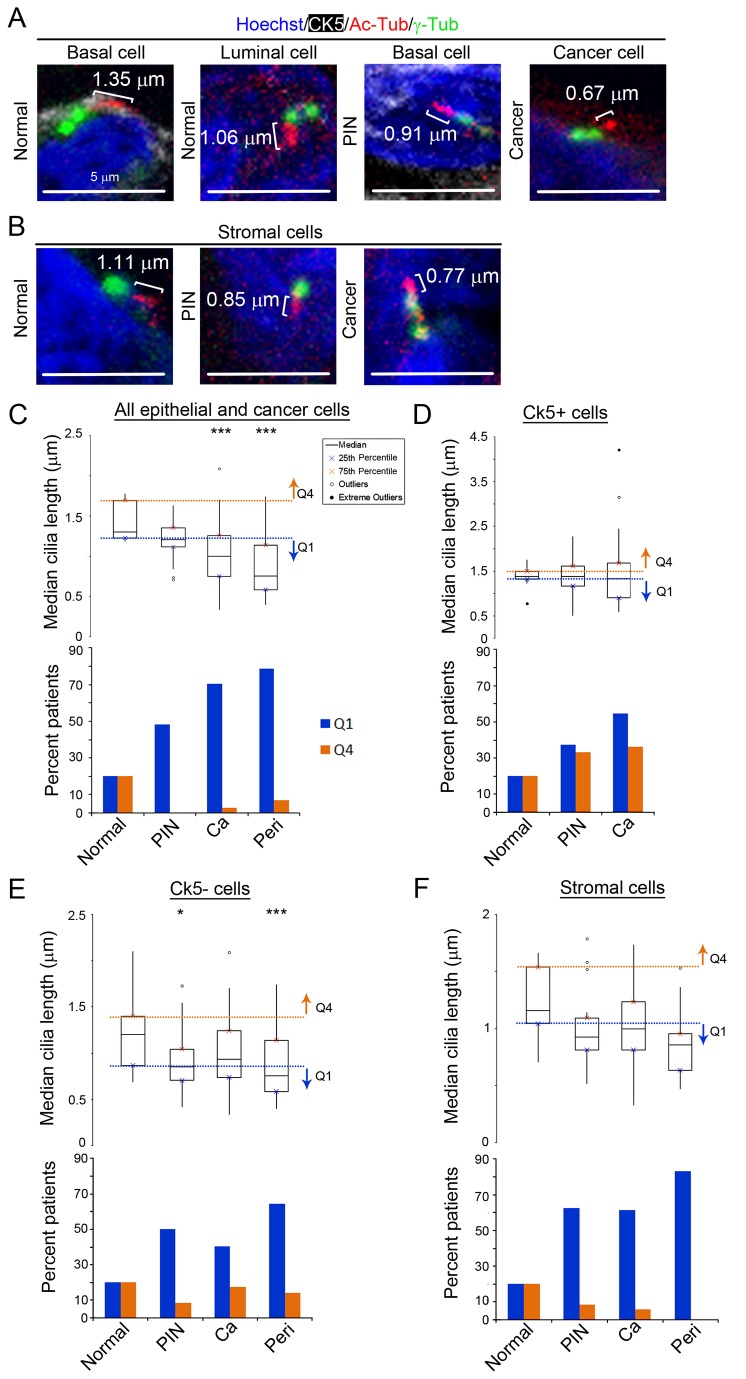
Ciliated prostate cancer and stromal cells have decreased axoneme lengths. (A and B) Primary cilia (Ac-Tub; red), centrosomes (γ-Tub; green), nuclei (Hoechst; blue), and cytokeratin 5 (CK5; white), are shown to illustrate representative ciliary lengths. Images were selected to represent the median length for normal and the 25^th^ percentile for PIN and cancer cells (note that many PIN and cancer cells have cilia shorter than those shown). Cilia lengths are given. (A) Normal basal cell, normal luminal cell, CK5+ basal cell in PIN, CK5-cancer cell and (B) stromal cell in normal, PIN, and cancer tissue. Boxplots of cilia lengths for (C, top) all epithelial, (D, top) CK5+, (E, top) CK5-and (F, top) stromal cells per patient for normal, PIN, cancer (Ca), and perineural invasion (Peri). Q4, Q1 are as in Figure 1. Statistics were performed using linear regression (* = p<0.05, *** = p<0.001) (C, D, E, and F, bottom). The percent of patients with abnormally long cilia (Q4; orange) or abnormally short cilia (Q1; blue).

Primary cilia lengths showed a small but not statistically significant decrease on preinvasive cells in PIN (median=1.22 µm; p=0.15) compared to normal (median=1.3 µm; Figure 4A, 4C, top and Table S4A in Table S4). However, the fraction of patients with abnormally short cilia increased in PIN (48.4%) compared to normal, suggesting that a subset of patients had shorter and therefore dysfunctional cilia (Figure 4C, bottom and Table S4B in Table S4). When the CK5+ and CK5-cells were analyzed separately, the only statistically significant decrease was observed in CK5-cells (median=0.85 µm; p=0.049) compared to normal (median=1.20 µm; Figure 4D, 4E, top and Table S4B in Table S4). The fraction of patients with abnormally short cilia increased on both CK5+ and CK5-cells in PIN (37.5% and 50%, respectively), suggesting cilia length shortens on both Ck5+ and Ck5-cells on PIN in a subset of patients (Figure 4D, 4E, bottom and Table S4B in Table S4).

Separating the PIN samples into LG and HG groups revealed a statistically significant difference in cilia lengths in HG PIN (median=1.21 µm; p=0.005) when compared to normal (median=1.30 µm; Table S4A in Table S4). In addition, the fraction of patients with abnormally short cilia increased in both LG and HG PIN (38.5% and 55.6% respectively) when compared to normal (Table S4B in Table S4). When the CK5+ and CK5-cell types were analyzed separately, the only statistically significant difference was observed in CK5-HG PIN (median=0.73 µm; p<0.0001) compared to CK5-normal (median=1.20 µm; Table S4A in Table S4). However, the fraction of patients with abnormally short cilia generally increased compared to normal in LG and HG PIN on both CK5+ and CK5-cells (CK5+: LG= 33.3%, HG=40%; CK5-: LG=22.2%, HG=66.7%). Collectively, this data suggest that a subpopulation of patients with LG and HG PIN have short and therefore dysfunctional cilia.

Analysis of all cancer cells demonstrated a statistically significant decrease in ciliary lengths in cancer (median=1.0 µm; p<0.0001) and perineural invasion lesions (median=0.76 µm; p<0.0001) compared to normal (median= 1.3 µm, Figure 4A, 4C, top and Table S4A in Table S4). Examining CK5+ and CK5-cancer cells separately revealed no statistically significant decrease in primary cilia length in the CK5+ or CK5-cancer cells (median=1.31 µm; p=0.77 and median=0.93 µm; p=0.093; Figure 4D, 4E, top and Table S4A in Table S4). This is likely due to decreased sample size. However, CK5+ and CK5-cancers cells both have an increased fraction of patients with abnormally short cilia compared to normal (54.5% and 40.5% respectively; Figure 4D and 4E, bottom and Table S4B in Table S4).

As with PIN, separating out the cancers into LG and HG groups revealed a statistically significant difference in cilia lengths. Interestingly, only LG cancers were seen to have significantly shorter cilia (median=0.86 µm; p<0.0001) compared to normal (median=1.3 µm), and this statistically significant difference was seen in both CK5+ cancer cells (median=0.93 µm; p<0.0001) as well as CK5-cancer cells (median=0.82 µm; p<0.0001; Table S4A in Table S4). However, the fraction of patients with abnormally short cilia lengths was generally increased in both LG and HG cancers on both CK5+ and CK5-cell types (CK5+: LG= 80%, HG=47.1%; CK5-: LG=54.3%, HG=28.2%; Table S4B in Table S4). It should be noted that although there was an approximately equal number of patients in the LG and HG cancer groups, all LG cancers were of Gleason sum score 6, and HG cancers were of Gleason sum score 7-10. The range of cancers with different Gleason sum scores in the HG cancer group, in conjunction with the low sample size (n=40), may explain the inability to reach a statistically significant difference between cilia lengths of HG cancer compared to normal. Together, this data demonstrates that when cilia are present in cancer samples, they are often short and possibly dysfunctional.

Analysis of cilia lengths on perineural invasion-associated cancer cells (median= 0.76 µm; p<0.0001) showed that primary cilia lengths had a statistically significant decrease compared to normal (median=1.3 µm; Figure 4C and Table S4A in Table S4). It should be noted that there were no cilia present on CK5+ perineural invasion cells and therefore shortening of cilia was only analyzed in the CK5-population. Collectively this data suggests that ciliary dysfunction, indicated by shortened ciliary length, persists in perineural invasive prostate cancer.

Statistical analysis of the linear trend from normal prostate progressing through PIN, cancer and then to perineural invasion was performed to determine if ciliary length decreases as prostate cancer severity increases. There was a statistically significant overall linear trend of decreasing cilia length with increasing severity of prostate cancer (p<0.0001; Table S4A in Table S4). Examining CK5+ and CK5-cells separately, there was a significant linear trend towards decreasing cilia length with increasing prostate cancer severity in the CK5-population (p=0.047) but not in the CK5+ population (p=0.998). These data further support the conclusion that ciliary dysfunction increases as prostate cancer progresses. This data may also suggest that CK5+ cells retain cilia that are of functional length, however, a larger sample size is needed. CK5-cells appear to have shortening of cilia consistent with ciliary dysfunction.

The lengths of stromal cell cilia were also determined (Figure 4F and Table S4A in Table S4). Compared to normal (median=1.16 µm), there was a small but not statistically significant decrease in cilia length on stromal cells surrounding PIN (median=0.93 µm; p=0.41), cancer (median=0.99 µm; p=0.72) and perineural invasion lesions (median=0.86 µm; p=0.25; Figure 4B and 4F, top). However, there was a clear increase in the fraction of patients with abnormally short cilia in PIN (62.5%), cancer (61.4%) and perineural invasion areas (83.3%; Figure 4F, bottom and Table S4B in Table S4) compared to normal. These data suggests that ciliary shortening may be an early event on stromal cells that contributes to prostate cancer development for a subset of patients. The different types of stromal cells are not differentiated so it is unclear if a certain cell type may be contributing to the overall shortening of cilia lengths observed in the stroma.

### Primary cilia loss correlates with increased nuclear β-catenin in normal prostate.

Given that cilia are known to suppress the canonical Wnt signaling pathway, we investigated if the loss of primary cilia in prostate cancer correlates with increased canonical Wnt signaling [2,3]. Nuclear β-catenin is a marker of activated canonical Wnt signaling [21]. Therefore, we measured nuclear β-catenin in the tissue types representative of increasingly severe stages of prostate cancer. We predicted that primary cilia loss or shortening would correlate with increased nuclear β-catenin. The tissue sections adjacent to the sections stained for primary cilia were stained for β-catenin and scored for percentage of positively stained nuclei and staining intensity (Figure S3).

**Figure 5:**
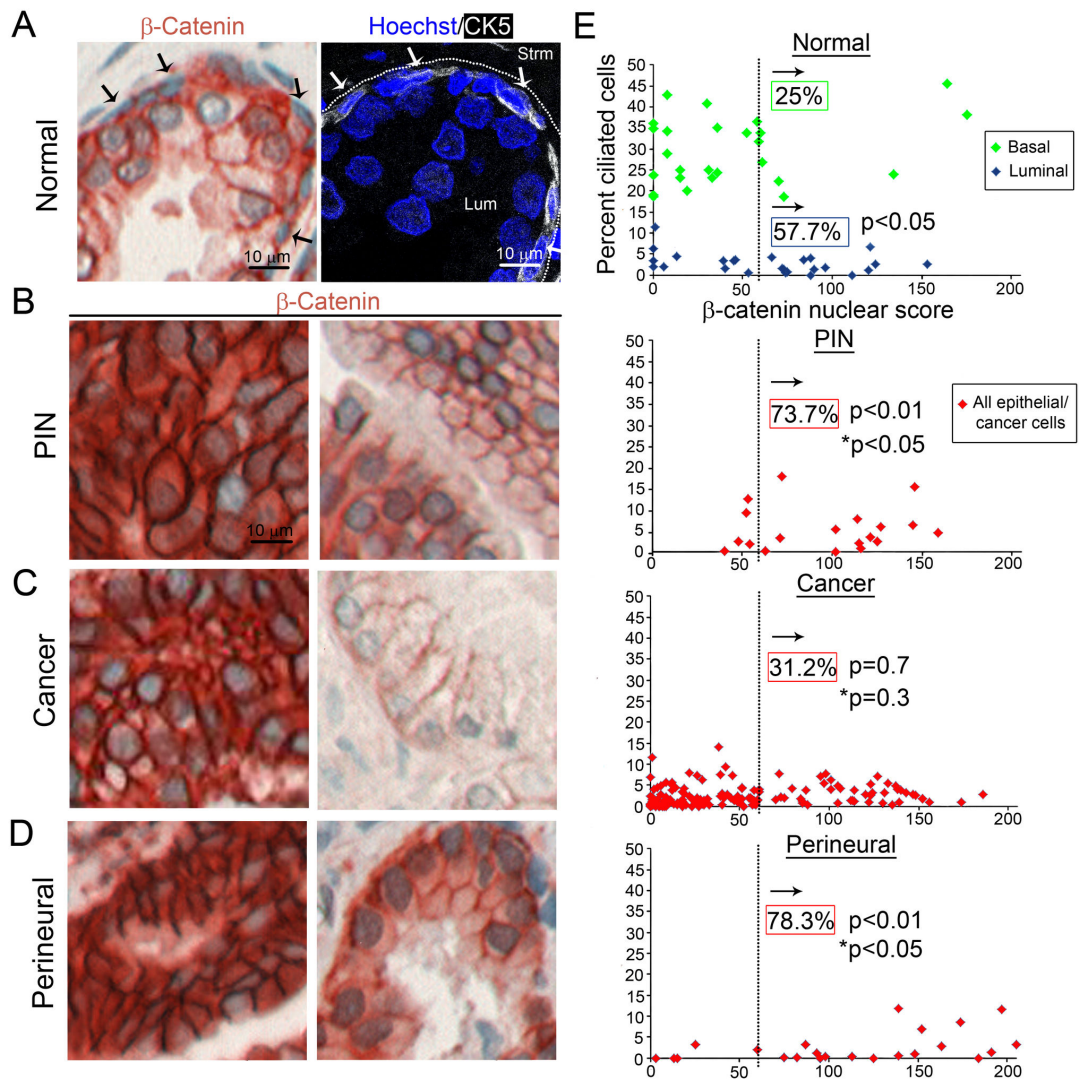
Primary cilia loss correlates with greater nuclear β-catenin in normal. (A) Immunohistochemistry (IHC) images of β-catenin staining in normal prostate, demonstrate a lack of β-catenin localization in basal cells (blue nuclei, black arrows; left). Basal cells were identified based on position, morphology, and cytokeratin 5 (CK5) staining in the adjoining serial section (white arrows; right). The lumen (Lum) and stroma (Strm) are labeled. IHC images show different levels and spatial distributions of β-catenin staining in (B) PIN, (C) cancer, and (D) perineural invasion. (E) Graphs display percent ciliated cells versus β-catenin nuclear score. Scattergraphs were plotted per location for normal, PIN, cancer, and perineural invasion tissue. For normal, basal cells (green) and luminal cells (blue) are shown. For PIN, cancer and perineural invasion, all epithelial/cancer cells are plotted red. High nuclear β-catenin was defined as the 75^th^ percentile of normal, denoted by the dotted line, and was used to compare normal to PIN, cancer and perineural cells. Percentages represent the locations with high nuclear β-catenin. Statistical analysis was performed using linear regression and included all of the data points, and comparisons to normal basal cells are denoted with p, and comparisons to normal luminal cells are denoted with p*.

In normal prostate tissue, luminal cells (correlating with low percentage of cilia) had higher nuclear β-catenin than basal cells (correlating with high percentage of cilia) (Figure 5A). 57.7% of luminal cells had high nuclear β-catenin scores (above the 75^th^ percentile of basal cells) (p<0.05; Figure 5E). Therefore, we found an inverse correlation between presence of cilia and nuclear β-catenin in normal prostate. This is consistent with cilia suppressing canonical Wnt signaling pathway in basal cells found in normal prostate tissue.

A wide range of β-catenin staining phenotypes was observed for PIN, cancer, and perineural invasion lesions (Figure 5B–5D). Based on staining in normal basal cells we set a cutoff for high nuclear β-catenin score as anything over the 75^th^ percentile of normal. The nuclear β-catenin score was high in 73.7% of PIN locations and this was statistically significant (p<0.01) compared to normal basal cells (Figure 5E). Comparison with normal luminal cells showed the nuclear β-catenin score was also significantly increased in PIN (p<0.05) (p* in Figure 5E). As shown previously, we observed a decrease in the percent ciliated cells in a subset of PIN (Figure 1C). This decrease in percent cilia in a subset of PIN correlates with an increase in nuclear β-catenin for a subset of patients.

In cancer locations, high nuclear β-catenin scores (31.2%) were not significantly different from normal basal (25%, p=0.7) or luminal cells (57.7%, p=0.3; Figure 5E). To determine if primary cilia in the cancers that had higher nuclear β-catenin were dysfunctional (as predicted by the ability of cilia to suppress the Wnt pathway), we added cilia lengths to this analysis. Although not statistically significant (p=0.10), likely due to insufficient sample size, the average cilia length was shorter for cancers with higher nuclear β-catenin (0.89 µm) compared to cancers with lower nuclear β-catenin (1.13 µm; Figure S4). This data suggests that cancers with shorter and therefore dysfunctional cilia have increased nuclear β-catenin.

Analysis of nuclear β-catenin in perineural invasion lesions showed high staining in 78.3% of perineural invasion locations (p<0.01) compared to normal basal cells. Comparison with normal luminal cells showed that nuclear β-catenin was again increased in perineural invasion lesions (p<0.05) (p* in Figure 5E). As shown previously (Figure 1C), perineural invasion lesions showed a significant loss of cilia. The increase in nuclear β-catenin correlates with loss of cilia. This is novel data, and may suggest canonical Wnt signaling as a mechanism which is activated to drive prostate cancer perineural invasion.

To determine if differences in nuclear β-catenin reflects differences in clinical data, we correlated clinical data with nuclear β-catenin scores by breaking up scores into low/moderate or high. The clinical data analyzed were: age at surgery, pathological tumor stage, pre-operative free PSA levels, biochemical recurrence, time to biochemical recurrence, capsular penetration, and largest tumor volume. Significant differences in incidence of capsular penetration and biochemical recurrence were found. A greater number of patients with high nuclear β-catenin had capsular penetration (40%; p<0.0001; Table S7) compared to patients with moderate/low nuclear β-catenin (2.6%). In contrast, a greater number of patients with lower nuclear β-catenin had biochemical recurrence (68.4%; p<0.0001) compared to patients with higher nuclear β-catenin (20%). Both presence of capsular penetration and biochemical recurrence are thought to be poor prognostic markers in prostate cancer. Our results demonstrate that high nuclear β-catenin correlates with capsular penetration but not with biochemical recurrence. It should be noted that for our cohort there are 38 patients in the moderate/low nuclear β-catenin group and 15 in the high nuclear β-catenin group (Table S7). In order to resolve these contradictory results, further analysis with a larger cohort of patients is needed.

### Evidence for a field effect influencing primary cilia frequency and length

Field effects in cancer, where normal cells adjacent to cancer cells are altered, have been documented [22]. To investigate if field effects on primary cilia expression and length exist in prostate cancer, we characterized primary cilia frequency and length in normal tissue from 16 cancerous prostates and compared it to normal tissue from the 10 patients without prostate cancer.

A slight increase in percent cilia was observed in normal epithelial cells adjacent to cancer (median=11.1%; p=0.257) compared to normal (median=8.9% Figure S5A and Table S5A in Table S5). Although this increase in percentage was not statistically significant, an increase in the proportion of patients with an abnormally high (greater than the 75^th^ percentile of normal) percent of ciliated cells was observed in normal tissue adjacent to cancer (43.8%; Figure S5C, bottom, and Table S5B in Table S5) and this increase was seen in both CK5+ and CK5-cells. For stromal cells, an increase in the proportion of patients with abnormally high percentages of ciliated stromal cells was also observed (43.8%; Figure S5D, bottom, and Table S5B in Table S5). Thus, there is a trend toward an increase in the percentage of ciliated cells in normal tissue adjacent to cancer.

Cilia lengths decreased slightly, although not significantly, in CK5+ (median=1.33 µm; p=0.975) and in CK5- (median=1.08 µm; p=0.79) epithelial cells compared to normal cells (median=1.38 µm and 1.2 µm respectively; Figure S6B-C, top and Table S6A in Table S6). A small, but not statistically significant, difference was seen for surrounding stromal cells (median=0.97 µm, p=0.596, Figure S6D, top, and Table S6A in Table S6). Despite the lack of significant differences, there was an increase in the fraction of patients with abnormally short cilia on all cell types; CK5+ epithelial (50%), CK5-epithelial (31.3%) and stromal (68.8%) cells (Figure S6A–D, bottom, and Table S6B in Table S6). Collectively, these data may reflect a potential field effect influencing primary cilia expression and length in normal tissue adjacent to cancer.

### Benign prostatic hyperplasia has altered primary cilia frequency and shortened primary cilia

Benign prostatic hyperplasia (BPH) is a common pathology in elderly men, with up to 50% of men 50 years old being diagnosed with this disease [23]. We characterized primary cilia expression and length in this non-malignant pathology.

The effects of BPH on frequency of primary cilia in the two different epithelial cell types were different. CK5+ ciliated cells displayed a slight decrease in ciliated cells (median=27.1%) compared to normal (median=28.8%; Figure S5B, top, and Table S5A in Table S5). Though the percentage ciliated cells was not statistically different (p=0.349), an increase in the proportion of patients with an abnormally low percent of ciliated CK5+ cells was observed (50%; Figure S5B, bottom, and Table S5B in Table S5). Conversely, CK5-ciliated cells were increased (median=6.1%) compared to normal (median=2.9%). Again, statistical significance was not reached (p=0.08; Figure S5C, top, and Table S5A in Table S5). However, 62.5% of patients had an abnormally high percent of ciliated CK5-cells (Figure S5C, bottom, and Table S5B in Table S5). For stromal cells, trends were observed suggesting a decrease in ciliated cells (median=4.3%; p=0.125) compared to normal (median=5.9%; Figure S5D, top, and Table S5A in Table S5). 50% of patients had an abnormally low percent of ciliated stromal cells (Figure S5D, bottom, and Table S5B in Table S5). Thus, in BPH, cilia numbers trend toward a decrease on CK5+ epithelial cells and stromal cells, but an increase on CK5-cells.

Cilia lengths were found to be decreased on both epithelial and stromal cells in BPH. On epithelial cells, cilia length significantly decreased (median=1.24 µm; p=0.048) compared to normal (median=1.3 µm; Figure S6A, top and Table S6A in Table S6). Although there was not a statistically significant decrease in cilia length on stromal cells (median=0.98 µm) compared to normal (median=1.16 µm), 75% of patients had abnormally short cilia compared to normal (Figure S6A and Table S6A, S6B in Table S6).

### Primary cilia frequency correlates with clinical data in normal tissue of a cancerous prostate and PIN

We next investigated if primary cilia frequency on epithelial cells from normal tissue adjacent to cancer, PIN, and prostate cancer tissues correlated with clinical data. The clinical data analyzed were: age at surgery, pathological tumor stage, pre-operative free PSA levels, biochemical recurrence, time to biochemical recurrence, capsular penetration, and largest tumor volume.

For normal tissue adjacent to cancer, statistically significant correlations were found for both CK5+ and CK5-epithelial cells, but not for the cell types combined (Tables S8, S9, and S10). A positive correlation was found for cilia frequency on CK5+ epithelial cells of normal tissue adjacent to cancer. Higher cilia frequency correlated with increasing tumor stage (p=0.037) and biochemical recurrence (p=0.04; Table S9). An inverse association was found for cilia frequency on CK5-epithelial cells in normal tissue adjacent to cancer. Lower frequency of cilia correlated with increased tumor size (p=0.015) and higher pre-operative free PSA (p=0.002; Table S10). For PIN, an inverse correlation was found for frequency of cilia on CK5+ epithelial cells. Lower percent of ciliated cells correlated with increased tumor size (p=0.006; Table S11). For both normal tissue adjacent to cancer and PIN, decreased cilia correlated with increasing tumor size. No significant correlations were found for cilia frequency in cancer (Table S12). It should be noted that the statistical analyses for cilia frequency and clinical data does not control for multiple comparisons.

## Discussion

This is the first study that has carefully characterized the frequency of primary cilia in human tissue types of different stages of prostate cancer. We have demonstrated that primary cilia are decreased early on epithelial cells in a subset of PIN lesions, and primary cilia are further reduced in cancers including perineural invasion lesions. We also show that primary cilia length is decreased in preinvasive and invasive prostate cancer, suggesting a potential dysfunctionality of the remaining cilia on preinvasive and malignant cells. The loss of primary cilia in preinvasive and invasive prostate cancer was correlated to the canonical Wnt signaling pathway, and we showed an association between primary cilia loss and increased nuclear β-catenin in normal tissue, and nuclear β-catenin is shown to be increased in PIN, a subset of cancers, and perineural invasion lesions. We also investigated associations between primary cilia frequency and clinical parameters, and most notably found that decreased cilia frequency correlated with increased tumor size.

Consistent with our findings in human prostate cancer tissue, Zhang et al. demonstrate that prostate cancer cell lines fail to express primary cilia *in vitro* [24]. Primary cilia in normal tissue adjacent to cancer, BPH, and prostate cancer were examined by electron microscopy in a study published in 1979 [25]. This study did not characterize cilia frequency in prostate cancer. However, it did demonstrate that the primary cilia expressed in prostate cancers often are of abnormal microtubule patterns, such as “7+3” or “9+2”, instead of the typical “9+0” pattern. Structural abnormalities of microtubule patterning in primary cilia likely affects signaling function. Taken together, these findings, along with data presented in this report, support the hypothesis that primary cilia are dysfunctional in prostate cancer either by being absent, shortened, or having abnormal microtubule structures.

We report low nuclear β-catenin, and therefore low canonical Wnt pathway activation, in the majority of invasive prostate cancers in our cohort (Figure 5E). There are numerous contradictory studies in the literature reporting on Wnt signaling activity measured by β-catenin staining in prostate cancer. Consistent with our report, some studies demonstrate absent or relatively low nuclear staining, and therefore low activation of the Wnt signaling pathway [26–30]. Other studies report an activated canonical Wnt signaling phenotype as a common event in prostate cancer [31–34]. Tissue fixation variations and differing primary antibody concentrations likely contribute to the differences observed [26]. Our results, which do not suggest Wnt signaling is activated in the majority of invasive prostate cancers, are surprising because we show the majority of prostate cancers have a loss of primary cilia (Figure 1). Primary cilia have been shown to suppress canonical Wnt signaling, therefore, the loss of cilia was hypothesized to result in increased nuclear β-catenin. This low nuclear β-catenin could be due to lack of canonical Wnt ligands in the tissue, or loss of pathway components needed to translocate β-catenin into the nucleus, such as Jouberin [3]. Interestingly, cancers surrounding nerves were shown to have increased nuclear β-catenin compared to other cancers (Figure 5E). Perhaps the microenvironment of perineural invasion preferentially allows for pathway activation. We did see a group of prostate cancers that had high nuclear β-catenin, and these cancers had both low percentage of ciliated cells and a decrease in cilia length (Figure S4). We hypothesize that shorter cilia may be dysfunctional and would fail to suppress the Wnt pathway. The low sample sizes of our cohort should be acknowledged. Our data suggests that other factors besides primary cilia may be controlling canonical Wnt signaling in the majority of prostate cancers.

Upon investigation of a correlation between clinical parameters (biochemical recurrence and capsular penetration) and nuclear β-catenin, we found that high nuclear β-catenin correlates with increased incidence of capsular penetration and that moderate/low β-catenin correlates with increased incidence of biochemical recurrence. These results appear to be contradictory given biochemical recurrence and capsular penetration have been associated with disease-recurrence [35–37]. However, several published reports on β-catenin agree with both of our results. Chen et al. demonstrated that higher total expression of β-catenin was found in higher tumor stage cancers (with capsular penetration) versus lower tumor stage cancers (no capsular penetration) [31]. Also in agreement with our findings, Horvath et al. investigated biochemical survival and correlated lower nuclear β-catenin with biochemical relapse [29]. These seemingly contradictory data, where high nuclear β-catenin correlated with capsular penetration and moderate/low nuclear β-catenin correlated with biochemical recurrence, may be explained by the biology of biochemical recurrence. Biochemical recurrence can occur as a result of PSA production by disseminated tumors cells that expand months following surgical removal of the primary tumor and this does not necessarily correlate with capsular penetration (pathological stage) which is a pathologic diagnosis made at the time of surgery [38]. This lack of correlation between biochemical recurrence and capsular penetration is also evident in our cohort. While this lack of correlation may explain our contradictory clinical correlations, further studies with a larger cohort are needed.

Our findings have shown that prostate cancer cells have a very low frequency of primary cilia. An important question for therapeutics is whether this loss of primary cilia is a driver of prostate cancer. A future direction is to determine if primary cilia loss is causal in tumor formation in the prostate. If cilia are causal in cancer formation, use of drugs that result in re-expression of primary cilia in cancers may be a promising therapy. This would require the determination of the cause(s) of primary cilia loss in prostate cancers. Many different mechanisms can result in cilia loss. There may be a loss of function of critical cilia genes required for proper construction and maintenance of primary cilia, through DNA mutations or chromosome loss. If loss of cilia is primarily caused by permanent genetic modulations, then re-expressing cilia may not be possible. Non-genetic drivers of cilia loss, such as signaling pathways or epigenetic drivers, could provide a more favorable therapeutic target. Alterations of signaling pathways have been shown to influence cilia expression. It has been shown that inhibiting Aurora-A-kinase can result in re-expression of primary cilia [39]. Interestingly, Aurora-A kinase over-expression has been shown to occur in prostate cancer [40]. Another pathway that could be targeted to allow for re-expression of primary cilia is the fatty acid synthesis pathway [41]. Fatty acid synthesis is common in prostate cancer, and blocking this pathway has been shown to cause re-expression of primary cilia in a prostate cancer cell line [41,42].

A decrease in primary cilia expression was observed in preinvasive lesions, specifically in a subset of LG and HG PIN. Although LG PIN is not generally thought to be a precursor to invasive prostate cancer, Goeman et al. demonstrate that in a patient with LG PIN in the initial biopsy there was a 30% risk of developing invasive cancer [43]. This was similar to the risk associated with a HG PIN diagnosis (27%) [43]. This data suggests that a subset of LG PIN have the potential to develop into invasive prostate cancer. We observed a decrease of primary cilia frequency (53.9% of patients; Figure S2B) and length (38.5% of patients; Figure S4B) in a subset of LG PIN samples, suggesting that these LG PIN lesions are similar to what is observed in HG PIN. It is possible that these LG PIN lesions with decreased cilia frequency may be precursors to prostate cancer formation. Further work is needed to determine if loss of cilia in LG PIN can be predictive of prostate cancer formation.

The frequency of primary cilia in cancers may be indicative of the efficacy of specific Hedgehog-targeted drugs for prostate cancer patients [44]. Numerous Hedgehog-targeted drugs are being investigated both in clinical trials and in laboratories. Most of these drugs inhibit Smoothened (Smo), a positive regulator of the pathway that requires primary cilia to be functional [44]. Since primary cilia are lost in prostate tumors, we hypothesize that prostate cancer patients would not be good candidates for Smo-inhibitor drugs. However, we demonstrate that primary cilia loss does not change in the stroma surrounding cancers (Figure 3). Karlou et al. have shown paracrine Hedgehog signaling in a prostate mouse model, where prostate cancer cells secrete Hedgehog ligand and Smo-dependent Hedgehog activity is restricted to the prostate tumor stroma [45]. We would predict that Smo-inhibitor drugs would be effective on the prostate cancer stroma, and may hinder the stroma-tumor relationship. However, treating a prostate cancer mouse model with a Smo-inhibitor did not reduce tumor burden, suggesting that Smo-inhibition may not be effective in prostate cancer patients [45].

In conclusion, primary cilia are decreased in a subset of preinvasive lesions, and are further lost in cancer and perineural invasion lesions. The rare tumor-associated primary cilia are shorter in preinvasive and invasive prostate cancer, which may reflect dysfunction. High nuclear β-catenin correlates with low primary cilia frequency in normal prostate tissue. In future work, it will be important to determine if primary cilia loss is causal to tumor formation.

## Supporting Information

Figure S1 Acetylated tubulin and Arl13b co-localize in the same primary cilia in normal and cancer tissues. Images show co-localization of two primary cilia markers in normal prostate tissue (top) and cancerous prostate tissue (bottom). Cells were stained for nuclei (Hoechst; blue) and centrosomes (γ-Tub; green) as well as with two commonly used cilia markers-- acetylated tubulin (Ac-Tub; red) and Arl13b (white).
(TIF)

Figure S2 Characterization of a rare population of CK5+ prostate cancer cells. (A) Cancerous prostate with a CK5 positive (CK5+) cell. H&E staining was used to identify a cancerous area that was stained for CK5 (white) and nuclei (Hoechst, blue) on the serially adjacent slide. (B) Percent of patients with cancers where no CK5+ cancer cells were identified (CK5-only cells; blue) and the percent of patients with cancers with at least one CK5+ cancer cell (red). (C) Percent of combined total epithelial or cancer cells that are CK5- (blue) and CK5+ (red) in normal, prostatic intraepithelial neoplasia (PIN), cancer and perineural invasion. (D) Percent of cancer cells that are CK5+ per patient. Data was plotted only for patients that had at least one CK5+ cell. (E) The αCK5 antibody used on the whole cohort (AbCam, cat # 24647) was compared to another commonly used Leica αCK5 antibody (Cat # CK5-R-7-CE) by staining serially adjacent normal tissue with each antibody.
(TIF)

Figure S3 Definiens Tissue Studio 3.0 software can classify nuclear β-catenin **staining intensity**. The process of image analysis for β-catenin using the Definiens Tissue Studio 3.0 software is shown for (A) normal and (B) cancer. From the TIFF image, the region of interest (ROI) is manually selected (blue/orange). The software identifies cells in the ROI based on selected parameters and thresholds which define the cell (green) and nucleus (blue). The nuclei are then classified as having no staining (white), low staining (yellow), medium staining (orange) or high staining (red). This information is used to compute the histological score (see methods).
(TIF)

Figure S4 Cancers with high nuclear β-catenin **have short cilia**. Percent ciliated cells is plotted versus β-catenin nuclear score (3X(% cells stained high) + 2X(% cells stained medium) +1X(% cells stained low)) versus cilia length (µm), where dot size reflects cilia lengths. The scattergraph was plotted per location, with data for all cancer cells. Cilia length key at bottom displays the normal range (the 25^th^ percentile of normal and the 75^th^ percentile of normal), the smallest and largest cilia lengths, and a cilia length directly between the smallest length and the normal 25^th^ percentile, and between the largest length and the 75^th^ normal percentile. The dotted line reflects the 75^th^ percentile of nuclear β-catenin scores for normal basal cells, which was used to define a high nuclear β-catenin score. The percentage denotes the percent of cancer locations with high nuclear β-catenin. Average cilia lengths for high nuclear β-catenin locations and moderate/low nuclear β-catenin locations are displayed.
(TIF)

Figure S5 Primary cilia frequency in benign structures adjacent to cancer. Percent of ciliated cells per patient for (A, top) all epithelial cells, (B, top) CK5+ epithelial cells (basal cells), (C, top) CK5-epithelial cells (luminal cells), and stromal cells (D, top) in normal tissue, normal tissue adjacent to cancer (N Adj. Ca), and benign prostatic hyperplasia (BPH). Q4, Q1 are as in Figure 1 (A-D, bottom). Percent of patients with an abnormally high percent cilia (Q4; orange), and an abnormally low percent cilia (Q1; blue) in (A, bottom) all epithelial cells, (B, bottom) CK5+ epithelial cells, (C, bottom) CK5-epithelial cells, and (D, bottom) stromal cells.
(TIF)

Figure S6 Primary cilia lengths in benign structures adjacent to cancer. Median cilia lengths for (A, top) all epithelial, (B, top) CK5+, (C, top) CK5-and (D, top) stromal cells per patient for each tissue type: normal, normal adjacent to cancer (N Adj. Ca), and benign prostatic hyperplasia (BPH). Q4 and Q1 are as in Figure 1. Statistical analysis was performed using linear regression. *,p<0.05 (A-D, bottom). The percent of patients with abnormally long cilia (Q4; orange) and abnormally short cilia (Q1; blue).
(TIF)

Table S1 Values for quantitation and analysis of percent ciliated epithelial/cancer cells in normal, PIN, cancer, and perineural. The data in this table corresponds to Figure 1 (Table S1A corresponds to Figure 1C boxplots, Table S1B corresponds to Figure 1C bar graphs). Figure 1C depicts boxplots of the percent of ciliated epithelial and cancer cells per patient for each tissue type: normal, prostatic intraepithelial neoplasia (PIN), cancer (Ca), and perinerual invasion (Peri). Bar graphs in Figure 1C depict the percent of patients with an abnormally high percent cilia (greater than the 75^th^ percentile for normal tissue ; Q4) or an abnormally low percent cilia (less than or equal to the 25^th^ percentile for normal tissue; Q1). Statistical analyses were not performed for LG and HG PIN and cancer separated, so no p-value was obtained for the individual grades.
(PDF)

Table S2 Values for quantitation and analysis of percent ciliated CK5+ and CK5-epithelial/cancer cells in normal, PIN, cancer, and perineural. The data in this table corresponds to Figure 2 (Table S2A corresponds to Figure 2C,D boxplots, Table S2B corresponds to Figure 2C,D bar graphs). Figure 2C,D depicts boxplots of the percent of ciliated CK5+ and CK5-epithelial and cancer cells per patient for each tissue type: normal, prostatic intraepithelial neoplasia (PIN), cancer (Ca), and perinerual invasion (Peri). Bar graphs in Figure 2C,D depict the percent of patients with an abnormally high percent cilia (greater than the 75^th^ percentile for normal tissue ; Q4) or an abnormally low percent cilia (less than or equal to the 25^th^ percentile for normal tissue; Q1). Statistical analyses were not performed for LG and HG PIN and cancer separated, so no p-value was obtained for the individual grades.
(PDF)

Table S3 Values for quantitation and analysis of percent ciliated stromal cells in normal, PIN, cancer and perineural. The data in this table corresponds to Figure 3 (Table S3A corresponds to Figure 3C boxplots, Table S3B corresponds to Figure 3C bar graphs). Figure 3C depicts boxplots of the percent of ciliated stromal cells per patient for each tissue type: normal, prostatic intraepithelial neoplasia (PIN), cancer (Ca), and perinerual invasion (Peri). Bar graphs in Figure 3C depict the percent of patients with an abnormally high percent cilia (greater than the 75^th^ percentile for normal tissue ; Q4) or an abnormally low percent cilia (less than or equal to the 25^th^ percentile for normal tissue; Q1). Statistical analyses were not performed for LG and HG PIN and cancer separated, so no p-value was obtained for the individual grades.
(PDF)

Table S4 Values for quantitation and analysis of cilia lengths in normal, PIN, cancer and perineural. The data in this table corresponds to Figure 4 (Table S4A corresponds to Figure 4C,D,E,F boxplots, Table S4B corresponds to Figure 4C,D,E,F bar graphs). Figure 4C,D,E,F depicts boxplots of median cilia lengths per patient broken up into cells type (all epithelial/cancer, CK5+, CK5-, stromal) for each tissue type: normal, prostatic intraepithelial neoplasia (PIN), cancer (Ca), and perinerual invasion (Peri). Bar graphs in Figure 4C,D,E,F depict the percent of patients with abnormally long cilia (greater than the 75^th^ percentile for normal tissue ; Q4) or abnormally short cilia (less than or equal to the 25^th^ percentile for normal tissue; Q1).
(PDF)

Table S5 Values for quantitation and analysis of percent ciliated epithelial/cancer and stromal cells in normal, normal adjacent to cancer, and BPH. The data in this table corresponds to Figure S5 (Table S5A corresponds to Figure S5A,B,C,D boxplots, Table S5B corresponds to Figure S5A,B,C,D bar graphs). Figure S5 depicts boxplots of the percent of ciliated cells per cell type (all epithelial/cancer, CK5+, CK5-, stromal) per patient for each tissue type: normal tissue, normal tissue adjacent to cancer (N Adj. Ca), and benign prostatic hyperplasia (BPH). Bar graphs in Figure S5A,B,C,D depict the percent of patients with an abnormally high percent cilia (greater than the 75^th^ percentile for normal tissue ; Q4) or an abnormally low percent cilia (less than or equal to the 25^th^ percentile for normal tissue; Q1).
(PDF)

Table S6 Values for quantitation and analysis of cilia lengths in normal, normal adjacent to cancer, and BPH. The data in this table corresponds to Figure S6 (Table S6A corresponds to Figure S6A,B,C,D boxplots, Table S6B corresponds to Figure S6A,B,C,D bar graphs). Figure S6 depicts boxplots of median cilia lengths per cell type (all epithelial/cancer, CK5+, CK5-, stromal) per patient for each tissue type: normal tissue, normal tissue adjacent to cancer (N Adj. Ca), and benign prostatic hyperplasia (BPH). Bar graphs in Figure S6A,B,C,D depict the percent of patients with abnormally long cilia (greater than the 75^th^ percentile for normal tissue ; Q4) or abnormally short cilia (less than or equal to the 25^th^ percentile for normal tissue; Q1).
(PDF)

Table S7 Statistically significant correlation between patient characteristics and high or moderate/low nuclear β-catenin in cancers. Nuclear β-catenin was correlated to capsular penetration and biochemical recurrence using a two-by-two table. Biochemical recurrence was defined as free serum PSA >0.1 ng/ml for two consecutive measurements. ≤75th percentile of nuclear β -catenin of normal basal cells is considered low/moderate nuclear β -catenin, while >75th percentile of nuclear β -catenin of normal basal cells is considered high nuclear β -catenin.
(PDF)

Table S8 Correlation between patient characteristics and percent cilia in all epithelial cells in normal adjacent to cancer. Patient characteristics were correlated to percent ciliated epithelial cells in normal tissue adjacent to cancer using linear regression. Number of patients =16.
(PDF)

Table S9 Correlation between patient characteristics and percent cilia in CK5+ epithelial cells in normal adjacent to cancer. Patient characteristics were correlated to percent ciliated CK5+ epithelial cells in normal tissue adjacent to cancer using linear regression. Number of patients =16.
(PDF)

Table S10 Correlation between patient characteristics and percent cilia in CK5-epithelial cells in normal adjacent to cancer. Patient characteristics were correlated to percent ciliated CK5-epithelial cells in normal tissue adjacent to cancer using linear regression. Number of patients =16.
(PDF)

Table S11 Correlation between patient characteristics and percent cilia in CK5+ PIN cells. Patient characteristics were correlated to percent ciliated CK5+ epithelial cells in PIN using linear regression. Number of patients =24.
(PDF)

Table S12 Correlation between patient characteristics and percent cilia in all cancer cells in prostate cancers. Patient characteristics were correlated to percent ciliated cancer cells using linear regression. Number of patients =75.
(PDF)

## References

[1] Pazour GJ, Agrin N, Leszyk J, Witman GB (2005) Proteomic analysis of a eukaryotic cilium. Journal of Cell Biology 170: 103-113. 15998802 10.1083/jcb.200504008 PMC2171396

[2] Corbit KC, Shyer AE, Dowdle WE, Gaulden J, Singla V et al. (2008) Kif3a constrains beta-catenin-dependent Wnt signalling through dual ciliary and non-ciliary mechanisms (vol 10, pg 70, 2008). Nat Cell Biol 10: 497-497. doi:10.1038/ncb0408-497a. 10.1038/ncb1670 18084282

[3] Lancaster MA, Schroth J, Gleeson JG (2011) Subcellular spatial regulation of canonical Wnt signalling at the primary cilium. Nat Cell Biol 13: 700-U173. PubMed: 21602792. 21602792 10.1038/ncb2259 PMC3107376

[4] Lancaster MA, Gleeson JG (2009) The primary cilium as a cellular signaling center: lessons from disease. Curr Opin Genet Dev 19: 220-229. doi:10.1016/j.gde.2009.04.008. PubMed: 19477114. 19477114 10.1016/j.gde.2009.04.008 PMC2953615

[5] Han YG, Kim HJ, Dlugosz AA, Ellison DW, Gilbertson RJ et al. (2009) Dual and opposing roles of primary cilia in medulloblastoma development. Nat Med 15: 1062-U1114. doi:10.1038/nm.2020. PubMed: 19701203. 19701203 10.1038/nm.2020 PMC2771737

[6] Wong SY, Seol AD, So PL, Ermilov AN, Bichakjian CK et al. (2009) Primary cilia can both mediate and suppress Hedgehog pathway-dependent tumorigenesis. Nat Med 15: 1055-U1109. doi:10.1038/nm.2011. PubMed: 19701205. 19701205 10.1038/nm.2011 PMC2895420

[7] Yuan K, Frolova N, Xie Y, Wang D, Cook L et al. (2010) Primary Cilia Are Decreased in Breast Cancer: Analysis of a Collection of Human Breast Cancer Cell Lines and Tissues. J Histochem Cytochem 58: 857-870. doi:10.1369/jhc.2010.955856. PubMed: 20530462. 20530462 10.1369/jhc.2010.955856 PMC2942739

[8] Schraml P, [!(surname)!], Thoma CR, Boysen G, Struckmann K, Krek W, Moch H (2009) Sporadic clear cell renal cell carcinoma but not the papillary type is characterized by severely reduced frequency of primary cilia. Mod Pathol 22: 31-36. PubMed: 18660794. 18660794 10.1038/modpathol.2008.132

[9] Kim J, Dabiri S, Seeley ES (2011) Primary Cilium Depletion Typifies Cutaneous Melanoma In Situ and Malignant Melanoma. PLOS ONE 6: e27410 PubMed: 22096570. 22096570 10.1371/journal.pone.0027410 PMC3214062

[10] Seeley ES, Carrière C, Goetze T, Longnecker DS, Korc M (2009) Pancreatic Cancer and Precursor Pancreatic Intraepithelial Neoplasia Lesions Are Devoid of Primary Cilia. Cancer Res 69: 422-430. doi:10.1158/0008-5472.CAN-08-1290. PubMed: 19147554. 19147554 10.1158/0008-5472.CAN-08-1290 PMC2629528

[11] SA G, BN R, PS B, BQ H, GB G, et al. (2013). p. HDAC6 inhibition restores ciliary expression and decreases tumor growth. Cancer Research 10.1158/0008-5472.CAN-12-2938 PMC3768151 23370327

[12] Epstein JI, Allsbrook WC, Amin MB, Egevad LL, Comm IG (2005) The 2005 International Society of Urological Pathology (ISUP) Consensus Conference on Gleason Grading of Prostatic Carcinoma. Am J Surg Pathol 29: 1228-1242. doi:10.1097/01.pas.0000173646.99337.b1. PubMed: 16096414. 16096414 10.1097/01.pas.0000173646.99337.b1

[13] Pan J, Snell W (2007) The primary cilium: Keeper of the key to cell division. Cell 129: 1255-1257. doi:10.1016/j.cell.2007.06.018. PubMed: 17604715. 17604715 10.1016/j.cell.2007.06.018

[14] Scholzen T, Gerdes J (2000) The Ki-67 protein: From the known and the unknown. J Cell Physiol 182: 311–22. PubMed: 10653597. 10653597 10.1002/(SICI)1097-4652(200003)182:3<311::AID-JCP1>3.0.CO;2-9

[15] Long RM, Morrissey C, Fitzpatrick JM, Watson RW (2005) Prostate epithelial cell differentiation and its relevance to the understanding of prostate cancer therapies. Clin Sci 108: 1–11. PubMed: 15384949. 15384949 10.1042/CS20040241

[16] Verhagen AP, Ramaekers FC, Aalders TW, Schaafsma HE, Debruyne FM et al. (1992) Colocalization of basal and luminal cell-type cytokeratins in human prostate cancer. Cancer Res 52: 6182–7. PubMed: 1384957. 1384957

[17] van Leenders GJ, Aalders TW, Hulsbergen-van de Kaa CA, Ruiter DJ, Schalken JA (2001) Expression of basal cell keratins in human prostate cancer metastases and cell lines. J Pathol 195: 563–70. PubMed: 11745692. 11745692 10.1002/path.993

[18] de Medina SGD, Salomon L, Colombel M, Abbou CC, Bellot J et al. (1998) Modulation of cytokeratin subtype, EGF receptor, and androgen receptor expression during progression of prostate cancer. Hum Pathol 29. 10.1016/s0046-8177(98)90208-8 9744319

[19] Hanahan D, Weinberg RA (2011) Hallmarks of Cancer: The Next Generation. Cell 144: 646–74. PubMed: 21376230. 21376230 10.1016/j.cell.2011.02.013

[20] Goetz SC, Anderson KV (2010) The primary cilium: a signalling centre during vertebrate development. Nat Rev Genet 11: 331-344. doi:10.1038/nrg2774. PubMed: 20395968. 20395968 10.1038/nrg2774 PMC3121168

[21] Nusse R (2005) Wnt signaling in disease and in development. Cell Res 15: 28-32. doi:10.1038/sj.cr.7290260. PubMed: 15686623. 15686623 10.1038/sj.cr.7290260

[22] Chai H, Brown RE (2009) Field Effect in Cancer-An Update. Ann Clin Lab Sci 39: 331-337. PubMed: 19880759. 19880759

[23] Kapoor A (2012) Benign prostatic hyperplasia (BPH) management in the primary care setting. Can J Urol 19 Suppl 1: 10–7. PubMed: 23089343. 23089343

[24] Zhang J, Lipinski RJ, Gipp JJ, Shaw AK, Bushman W (2009) Hedgehog pathway responsiveness correlates with the presence of primary cilia on prostate stromal cells. BMC Dev Biol 9: 50-. PubMed: 19811645. 19811645 10.1186/1471-213X-9-50 PMC2767347

[25] Ohtsuki Y, Dmochowski L (1979) Fine structure of single cilium in human prostatic neoplasia. J Clin Electron Microsc 12: 81-90.

[26] Whitaker HC, Girling J, Warren AY, Leung H, Mills IG et al. (2008) Alterations in beta-catenin expression and localization in prostate cancer. Prostate 68: 1196-1205. doi:10.1002/pros.20780. PubMed: 18459111. 18459111 10.1002/pros.20780

[27] Assikis VJ, Do KA, Wen S, Wang X, Cho-Vega JH et al. (2004) Clinical and biomarker correlates of androgen-independent, locally aggressive prostate cancer with limited metastatic potential. Clin Cancer Res 10: 6770-6778. doi:10.1158/1078-0432.CCR-04-0275. PubMed: 15501953. 15501953 10.1158/1078-0432.CCR-04-0275

[28] Arenas MI, Romo E, Royuela M, Fraile B, Paniagua R (2000) E-, N- and P-cadherin, and alpha-, beta- and gamma-catenin protein expression in normal, hyperplastic and carcinomatous human prostate. Histochem J 32: 659-667. doi:10.1023/A:1004111331752. PubMed: 11272805. 11272805 10.1023/a:1004111331752

[29] Horvath LG, Henshall SM, Lee CS, Kench JG, Golovsky D et al. (2005) Lower levels of nuclear beta-catenin predict for a poorer prognosis in localized prostate cancer. Int J Cancer 113: 415-422. doi:10.1002/ijc.20599. PubMed: 15455387. 15455387 10.1002/ijc.20599

[30] Bismar TA, Humphrey PA, Grignon DJ, Wang HL (2004) Expression of beta-catenin in prostatic adenocarcinomas: A comparison with colorectal adenocarcinomas. Am J Clin Pathol 121: 557-563. doi:10.1309/447049GV52H7D258. PubMed: 15080308. 15080308 10.1309/4470-49GV-52H7-D258

[31] Chen G, Shukeir N, Potti A, Sircar K, Aprikian A et al. (2004) Up-regulation of Wnt-1 and beta-catenin production in patients with advanced metastatic prostate carcinoma: Potential pathogenetic and prognostic implications. Cancer 101: 1345-1356. doi:10.1002/cncr.20518. PubMed: 15316903. 15316903 10.1002/cncr.20518

[32] Aaltomaa S, Kärjä V, Lipponen P, Isotalo T, Kankkunen JP et al. (2005) Reduced alpha- and beta-catenin expression predicts shortened survival in local prostate cancer. Anticancer Res 25: 4707–12. PubMed: 16334164. 16334164

[33] Morita N, Uemura H, Tsumatani K, Cho M, Hirao Y et al. (1999) E-cadherin and alpha-, beta- and gamma-catenin expression in prostate cancers: correlation with tumour invasion. Br J Cancer 79: 1879–83. PubMed: 10206308. 10206308 10.1038/sj.bjc.6690299 PMC2362820

[34] Jaggi M, Johansson SL, Baker JJ, Smith LM, Galich A et al. (2005) Aberrant expression of E-cadherin and beta-catenin in human prostate cancer. Urol Oncol 23: 402-406. doi:10.1016/j.urolonc.2005.03.024. PubMed: 16301117. 16301117 10.1016/j.urolonc.2005.03.024

[35] Theiss M, Wirth MP, Manseck A, Frohmüller HG (1995) Prognostic significance of capsular invasion and capsular penetration in patients with clinically localized prostate cancer undergoing radical prostatectomy. Prostate 27: 13–7. PubMed: 7603912. 7603912 10.1002/pros.2990270104

[36] Freedland SJ, Humphreys EB, Mangold LA, Eisenberger M, Dorey FJ et al. (2005) Risk of prostate cancer-specific mortality following biochemical recurrence after radical prostatectomy. JAMA 294: 433–9. PubMed: 16046649. 16046649 10.1001/jama.294.4.433

[37] Sakar I, Harada K-I, Hara I, Eto H, Miyake H (2004) Prognostic Significance of Capsular Invasion and Capsular Penetration in Japanese Men with Prostate Cancer Undergoing Radical Retropubic Prostatectomy. Urol Oncol 4: 27-30.

[38] Morgan TM, Lange PH, Porter MP, Lin DW, Ellis WJ et al. (2009) Disseminated Tumor Cells in Prostate Cancer Patients after Radical Prostatectomy and without Evidence of Disease Predicts Biochemical Recurrence. Clin Cancer Res 15: 677-683. doi:10.1158/1078-0432.CCR-08-1754. PubMed: 19147774. 19147774 10.1158/1078-0432.CCR-08-1754 PMC3162324

[39] Egeberg DL, Lethan M, Manguso R, Schneider L, Awan A et al. (2012) Primary cilia and aberrant cell signaling in epithelial ovarian cancer. Cilia 1: 15-. PubMed: 23351307. 23351307 10.1186/2046-2530-1-15 PMC3555760

[40] Buschhorn HM, Klein RR, Chambers SM, Hardy MC, Green S et al. (2005) Aurora-A over-expression in high-grade PIN lesions and prostate cancer. Prostate 64: 341–6. PubMed: 15754349. 15754349 10.1002/pros.20247

[41] Willemarck N, Rysman E, Brusselmans K, Van Imschoot G, Vanderhoydonc F et al. (2010) Aberrant Activation of Fatty Acid Synthesis Suppresses Primary Cilium Formation and Distorts Tissue Development. Cancer Res 70: 9453–62. PubMed: 20889723. 20889723 10.1158/0008-5472.CAN-10-2324

[42] Schmidt LJ, Ballman KV, Tindall DJ (2007) Inhibition of fatty acid synthase activity in prostate cancer cells by dutasteride. Prostate 67: 1111-1120. doi:10.1002/pros.20602. PubMed: 17477363. 17477363 10.1002/pros.20602

[43] Goeman L, Joniau S, Ponette D, Van der Aa F, Roskams T et al. (2003) Is low-grade prostatic intraepithelial neoplasia a risk factor for cancer? Prostate Cancer Prostatic Dis 6: 305-310. doi:10.1038/sj.pcan.4500681. PubMed: 14663472. 14663472 10.1038/sj.pcan.4500681

[44] Hassounah NB, Bunch TA, McDermott KM (2012) Molecular Pathways: The Role of Primary Cilia in Cancer Progression and Therapeutics with a Focus on Hedgehog Signaling. Clin Cancer Res 18: 2429-2435. doi:10.1158/1078-0432.CCR-11-0755. PubMed: 22415315. 22415315 10.1158/1078-0432.CCR-11-0755 PMC3738179

[45] Karlou M, Lu JF, Wu G, Maity S, Tzelepi V et al. (2012) Hedgehog signaling inhibition by the small molecule smoothened inhibitor GDC-0449 in the bone forming prostate cancer xenograft MDA PCa 118b. Prostate 72: 1638–47. PubMed: 22457212. 22457212 10.1002/pros.22517 PMC4977841

