# Supplementary figures and images for: Primary Cilia Are Lost in Preinvasive and Invasive Prostate Cancer
Source: PLoS One. 2013 Jul 2;8(7):e68521. doi: 10.1371/journal.pone.0068521 (PMC3699526; doi:10.1371/journal.pone.0068521)

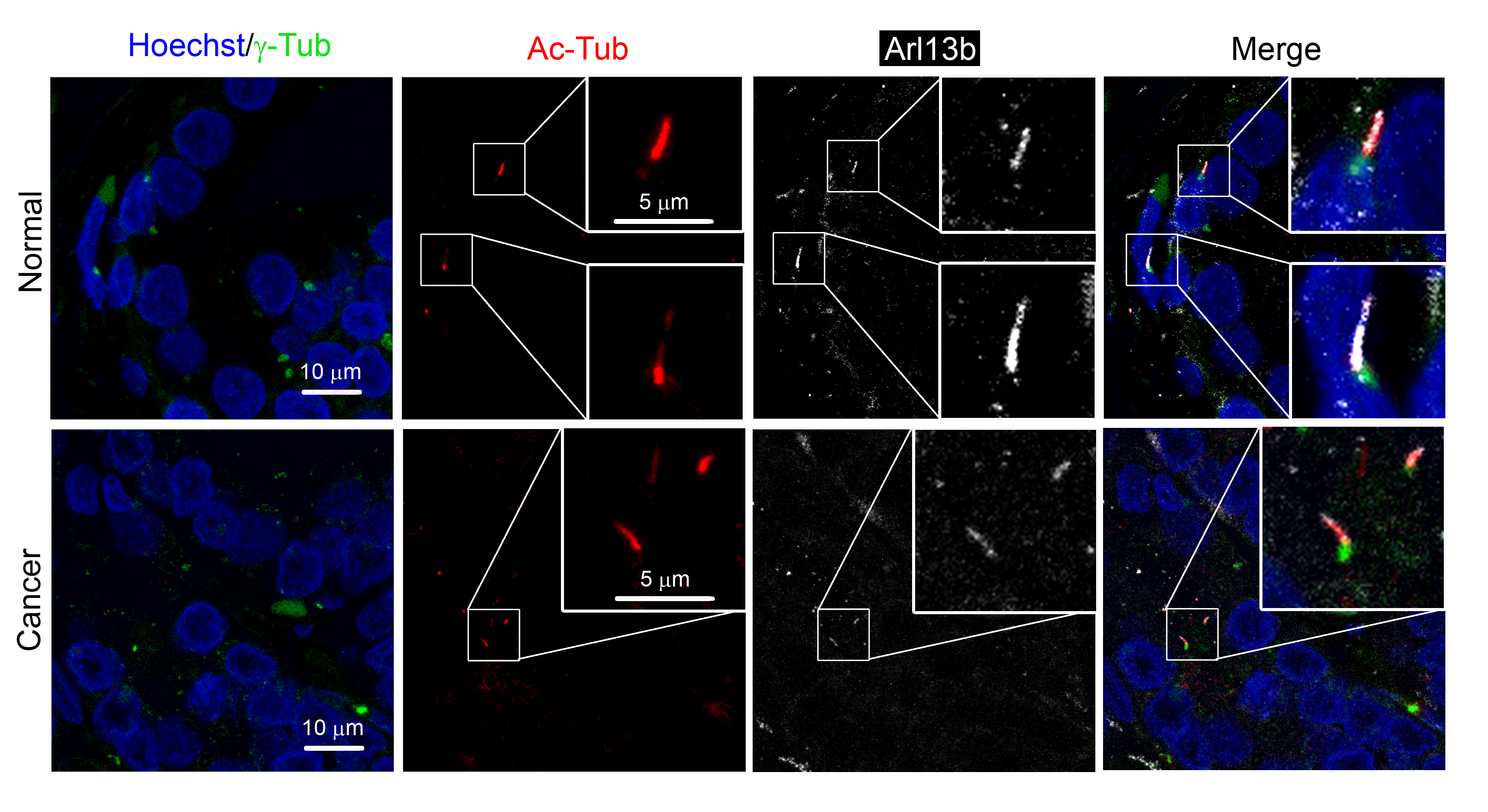

Supplement: Figure S1 — Images show co-localization of two primary cilia markers in normal prostate tissue (top) and cancerous prostate tissue (bottom). Cells were stained for nuclei (Hoechst; blue) and centrosomes (γ-Tub; green) as well as with two commonly used cilia markers-- acetylated tubulin (Ac-Tub; red) and Arl13b (white). (TIF) [file pone.0068521.s001.tif]

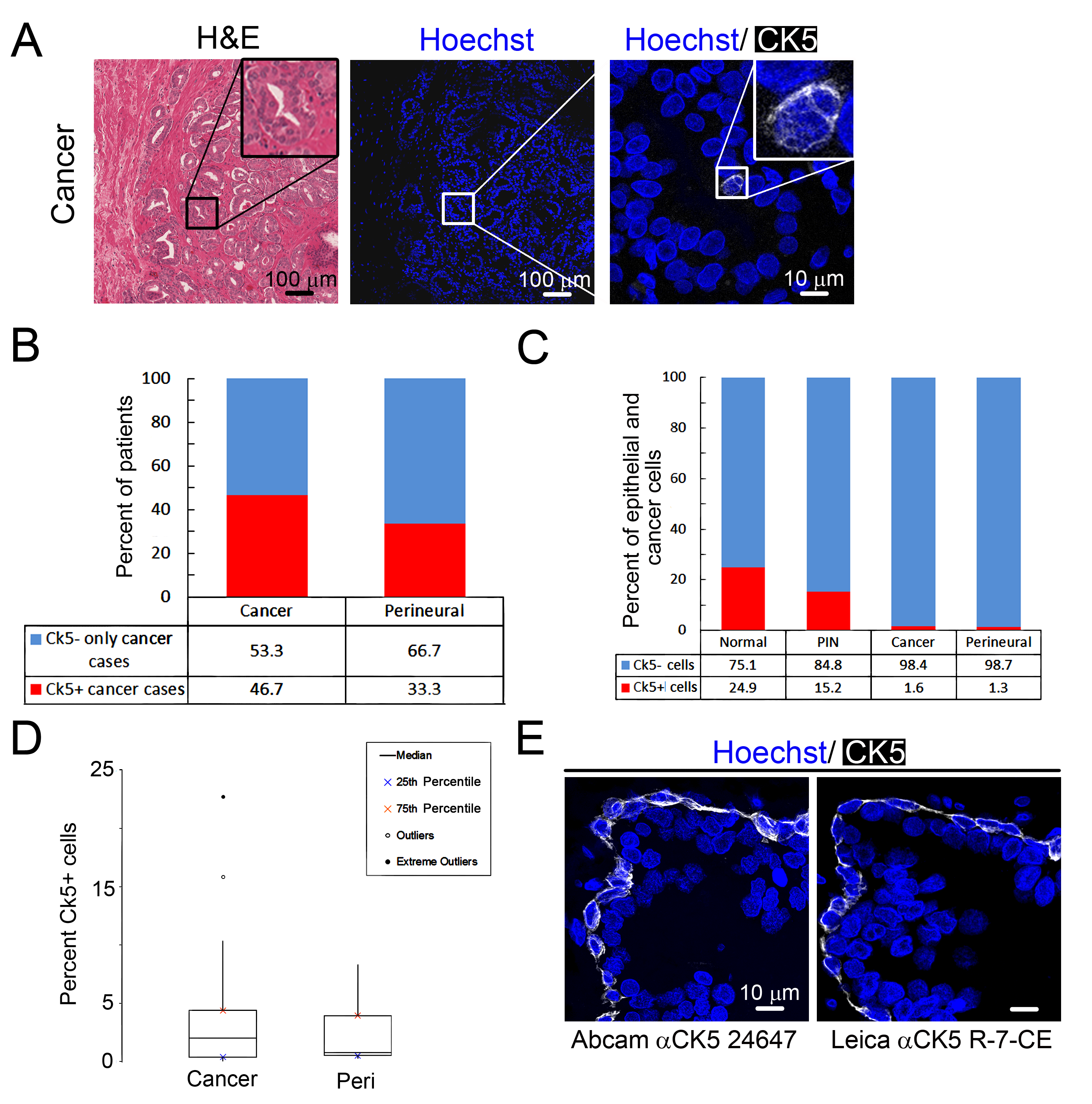

Supplement: Figure S2 — (A) Cancerous prostate with a CK5 positive (CK5+) cell. H&E staining was used to identify a cancerous area that was stained for CK5 (white) and nuclei (Hoechst, blue) on the serially adjacent slide. (B) Percent of patients with cancers where no CK5+ cancer cells were identified (CK5-only cells; blue) and the percent of patients with cancers with at least one CK5+ cancer cell (red). (C) Percent of combined total epithelial or cancer cells that are CK5- (blue) and CK5+ (red) in normal, prostatic intraepithelial neoplasia (PIN), cancer and perineural invasion. (D) Percent of cancer cells that are CK5+ per patient. Data was plotted only for patients that had at least one CK5+ cell. (E) The αCK5 antibody used on the whole cohort (AbCam, cat # 24647) was compared to another commonly used Leica αCK5 antibody (Cat # CK5-R-7-CE) by staining serially adjacent normal tissue with each antibody. (TIF) [file pone.0068521.s002.tif]

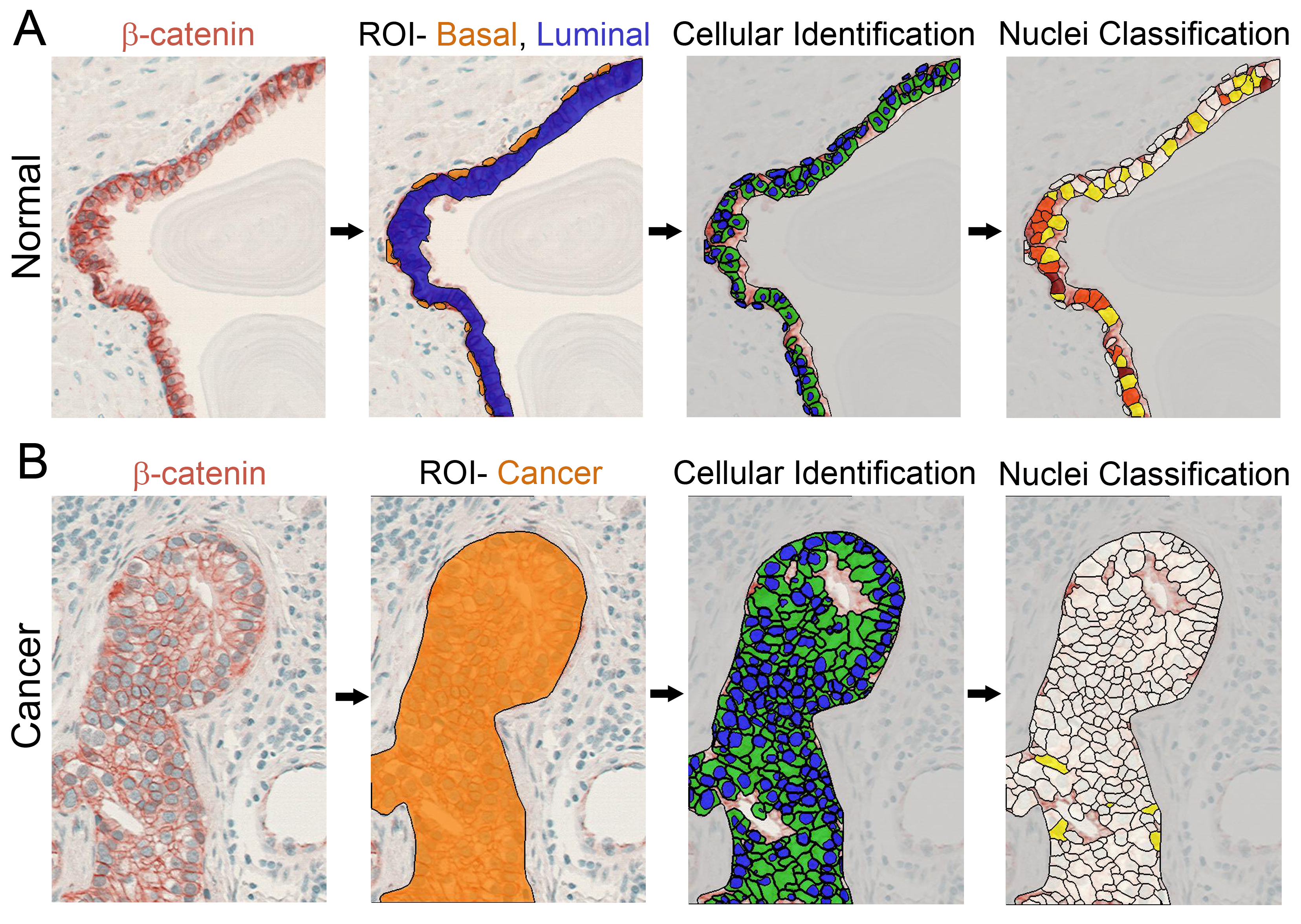

Supplement: Figure S3 — β-catenin staining intensity. The process of image analysis for β-catenin using the Definiens Tissue Studio 3.0 software is shown for (A) normal and (B) cancer. From the TIFF image, the region of interest (ROI) is manually selected (blue/orange). The software identifies cells in the ROI based on selected parameters and thresholds which define the cell (green) and nucleus (blue). The nuclei are then classified as having no staining (white), low staining (yellow), medium staining (orange) or high staining (red). This information is used to compute the histological score (see methods). (TIF) [file pone.0068521.s003.tif]

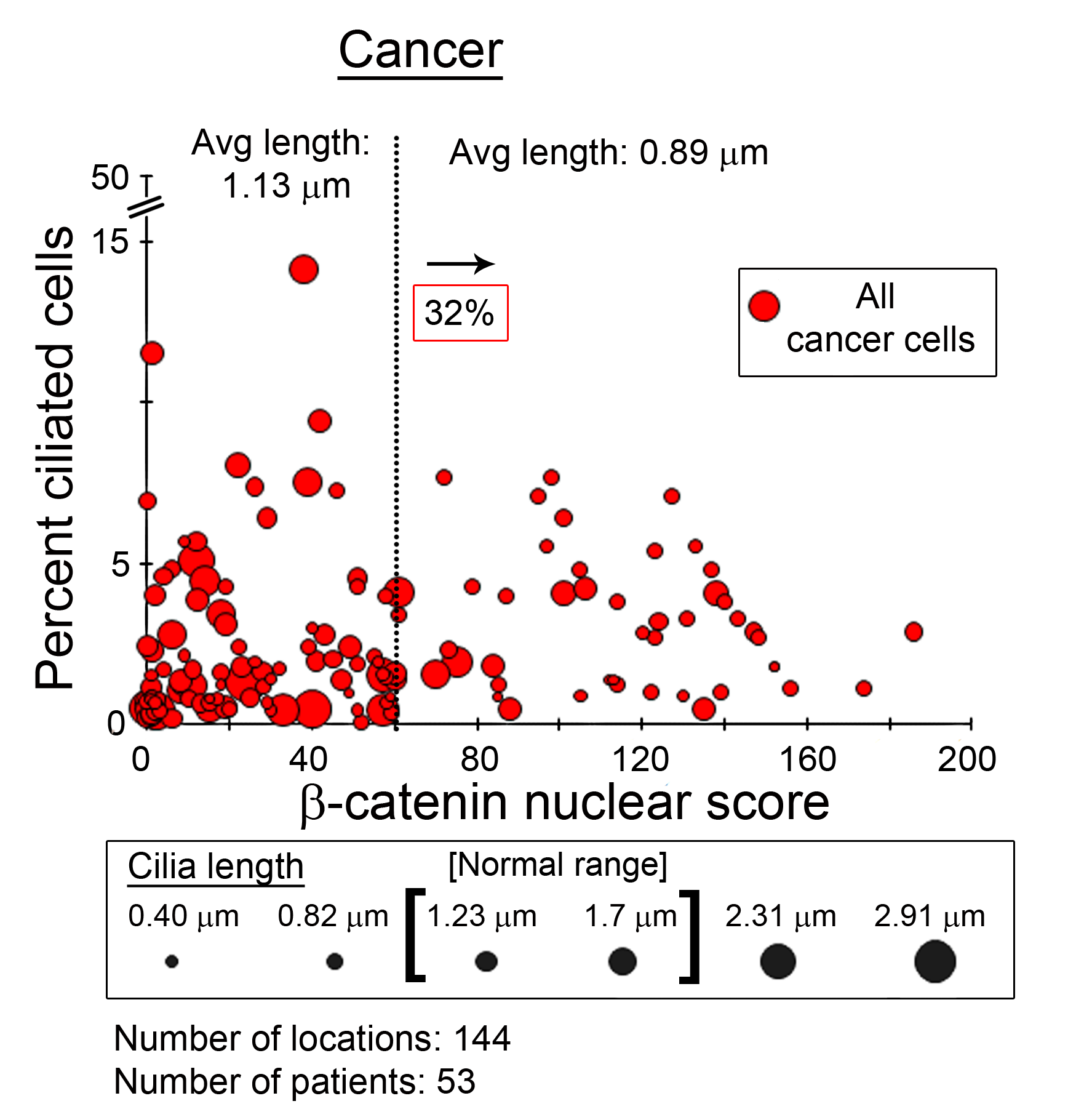

Supplement: Figure S4 — β-catenin have short cilia. Percent ciliated cells is plotted versus β-catenin nuclear score (3X(% cells stained high) + 2X(% cells stained medium) +1X(% cells stained low)) versus cilia length (µm), where dot size reflects cilia lengths. The scattergraph was plotted per location, with data for all cancer cells. Cilia length key at bottom displays the normal range (the 25th percentile of normal and the 75th percentile of normal), the smallest and largest cilia lengths, and a cilia length directly between the smallest length and the normal 25th percentile, and between the largest length and the 75th normal percentile. The dotted line reflects the 75th percentile of nuclear β-catenin scores for normal basal cells, which was used to define a high nuclear β-catenin score. The percentage denotes the percent of cancer locations with high nuclear β-catenin. Average cilia lengths for high nuclear β-catenin locations and moderate/low nuclear β-catenin locations are displayed. (TIF) [file pone.0068521.s004.tif]

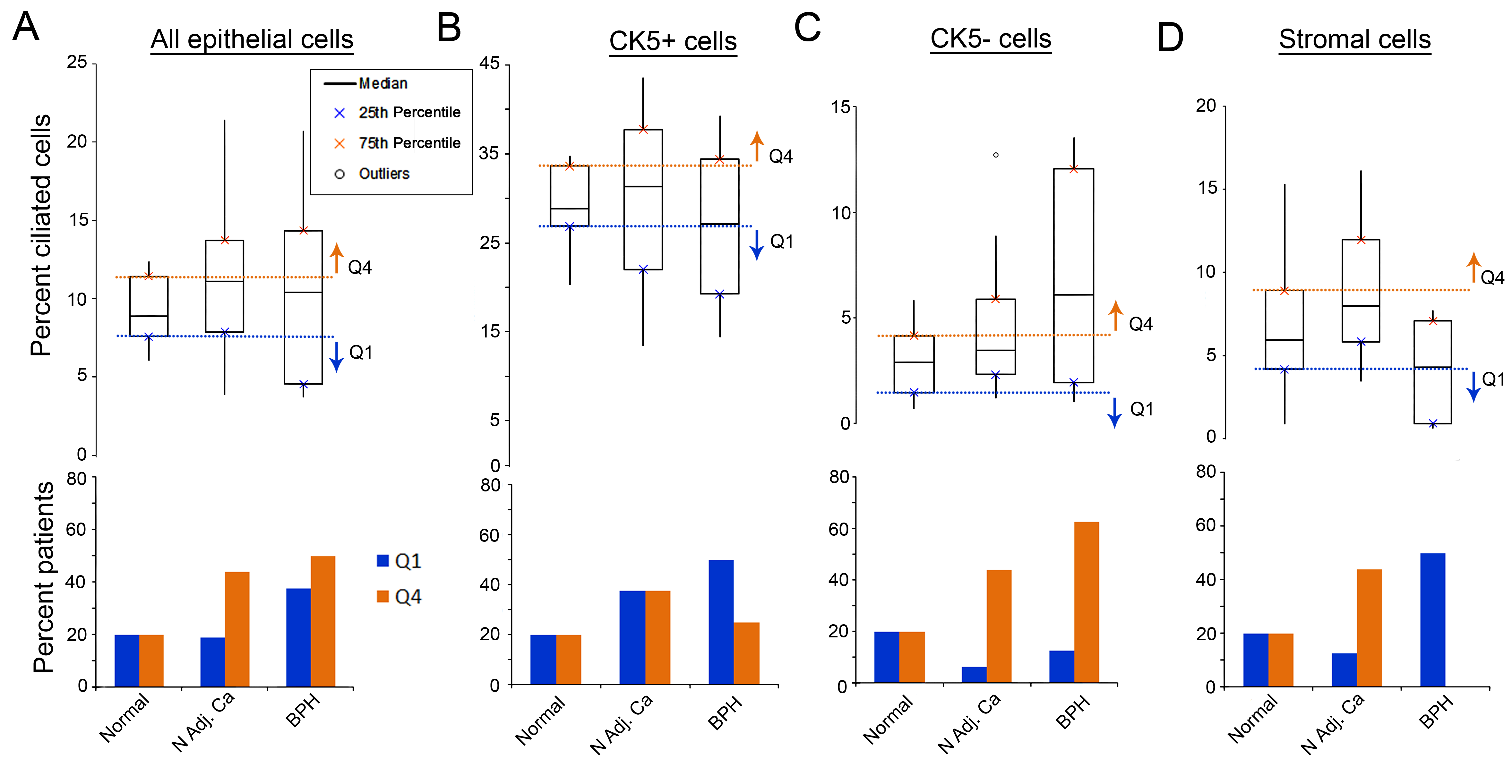

Supplement: Figure S5 — Percent of ciliated cells per patient for (A, top) all epithelial cells, (B, top) CK5+ epithelial cells (basal cells), (C, top) CK5-epithelial cells (luminal cells), and stromal cells (D, top) in normal tissue, normal tissue adjacent to cancer (N Adj. Ca), and benign prostatic hyperplasia (BPH). Q4, Q1 are as in Figure 1 (A-D, bottom). Percent of patients with an abnormally high percent cilia (Q4; orange), and an abnormally low percent cilia (Q1; blue) in (A, bottom) all epithelial cells, (B, bottom) CK5+ epithelial cells, (C, bottom) CK5-epithelial cells, and (D, bottom) stromal cells. (TIF) [file pone.0068521.s005.tif]

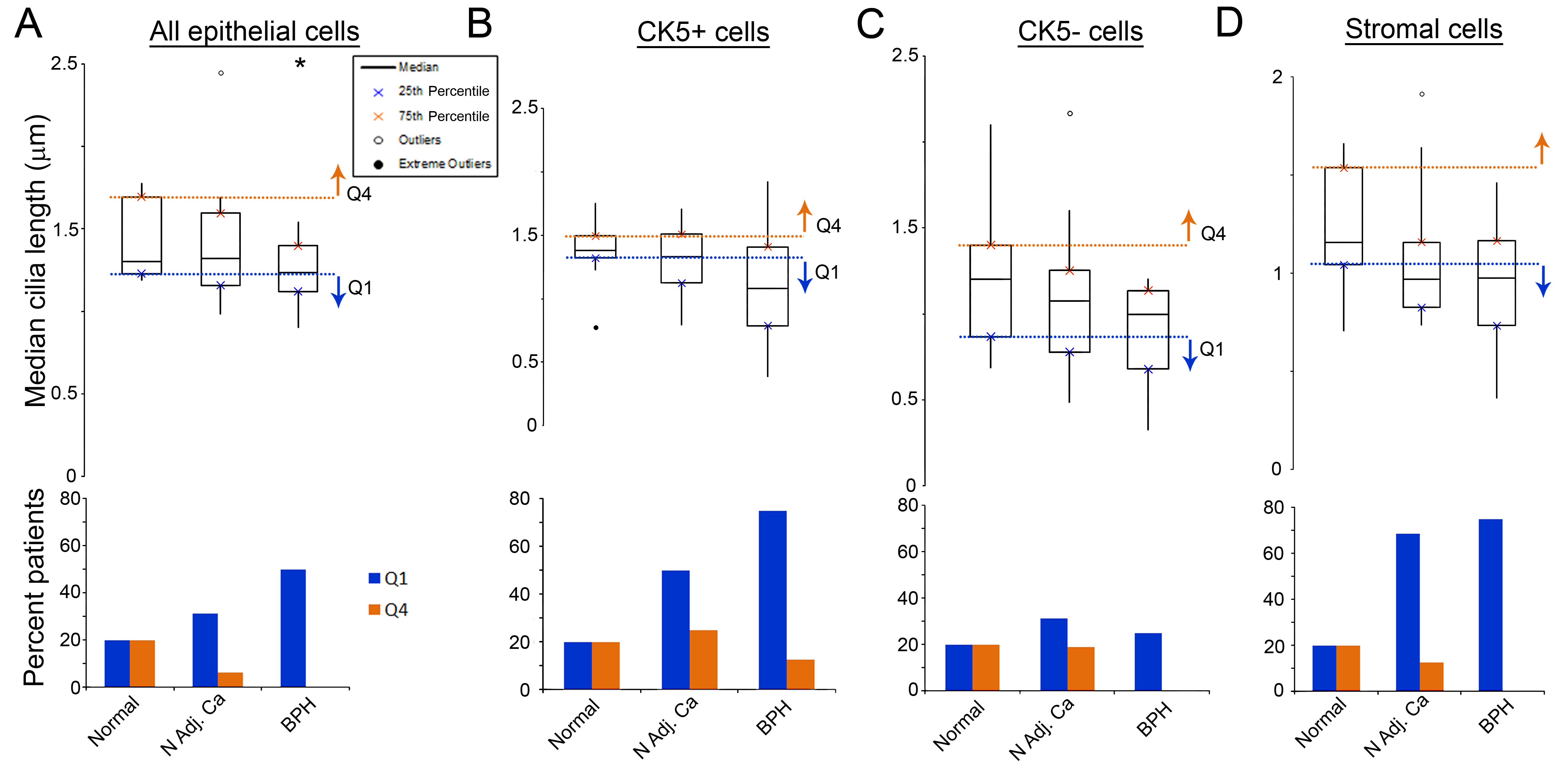

Supplement: Figure S6 — Median cilia lengths for (A, top) all epithelial, (B, top) CK5+, (C, top) CK5-and (D, top) stromal cells per patient for each tissue type: normal, normal adjacent to cancer (N Adj. Ca), and benign prostatic hyperplasia (BPH). Q4 and Q1 are as in Figure 1. Statistical analysis was performed using linear regression. *,p<0.05 (A-D, bottom). The percent of patients with abnormally long cilia (Q4; orange) and abnormally short cilia (Q1; blue). (TIF) [file pone.0068521.s006.tif]
