# Supplementary material for: Primary Cilia Are Lost in Preinvasive and Invasive Prostate Cancer
Source: PLoS One. 2013 Jul 2;8(7):e68521. doi: 10.1371/journal.pone.0068521 (PMC3699526; doi:10.1371/journal.pone.0068521)
Supplement: Table S1 — The data in this table corresponds to Figure 1 (Table S1A corresponds to Figure 1C boxplots, Table S1B corresponds to Figure 1C bar graphs). Figure 1C depicts boxplots of the percent of ciliated epithelial and cancer cells per patient for each tissue type: normal, prostatic intraepithelial neoplasia (PIN), cancer (Ca), and perinerual invasion (Peri). Bar graphs in Figure 1C depict the percent of patients with an abnormally high percent cilia (greater than the 75th percentile for normal tissue ; Q4) or an abnormally low percent cilia (less than or equal to the 25th percentile for normal tissue; Q1). Statistical analyses were not performed for LG and HG PIN and cancer separated, so no p-value was obtained for the individual grades. (PDF) [file pone.0068521.s007.pdf]

Table S1A: Values for quantitation of percent ciliated epithelial/cancer cells in normal, PIN, cancer, and perineural.

**Boxplot all epithelial/cancer cells**						
	n (patients)	% cilia median	n (total nuclei)	Range nuclei/patient	Range cilia/patient	P-value
Normal	10	8.9	6517	405-1097	25-95	n/a
PIN	24	5.7	22951	325-1759	8-172	0.24
PIN LG	13	6.6	9652	325-1698	17-172	n/a
PIN HG	18	5.8	13299	378-1759	8-102	n/a
Ca	75	1.9	52877	112-1923	0-69	**<0.0001**
Ca LG	35	2.2	18973	208-1273	1-49	n/a
Ca HG	40	1.4	33904	112-1923	0-69	n/a
Peri	18	1.7	8082	120-1393	0-72	**<0.0001**
Average	29	4.3	20782	248-1603	7-100	trend**<0.0001**

Table S1B: Values for analysis of percent ciliated epithelial/cancer cells in normal, PIN, cancer, and perineural.

**Bar graph all epithelial/cancer cells**				
	Q1 n(patients)	Q1 % (patients)	Q4 n (patients)	Q4 % (patients)
Normal	2	20	2	20
PIN	17	70.8	3	12.5
PIN LG	8	61.5	2	15.4
PIN HG	14	77.8	2	11.1
Ca	74	98.7	0	0
Ca LG	35	100	0	0
Ca HG	39	97.5	0	0
Peri	17	94.4	1	5.6
Q1 ≤ 7.6%, Q4 >11.4%				
