# Supplementary material for: Primary Cilia Are Lost in Preinvasive and Invasive Prostate Cancer
Source: PLoS One. 2013 Jul 2;8(7):e68521. doi: 10.1371/journal.pone.0068521 (PMC3699526; doi:10.1371/journal.pone.0068521)
Supplement: Table S2 — The data in this table corresponds to Figure 2 (Table S2A corresponds to Figure 2C,D boxplots, Table S2B corresponds to Figure 2C,D bar graphs). Figure 2C,D depicts boxplots of the percent of ciliated CK5+ and CK5-epithelial and cancer cells per patient for each tissue type: normal, prostatic intraepithelial neoplasia (PIN), cancer (Ca), and perinerual invasion (Peri). Bar graphs in Figure 2C,D depict the percent of patients with an abnormally high percent cilia (greater than the 75th percentile for normal tissue ; Q4) or an abnormally low percent cilia (less than or equal to the 25th percentile for normal tissue; Q1). Statistical analyses were not performed for LG and HG PIN and cancer separated, so no p-value was obtained for the individual grades. (PDF) [file pone.0068521.s008.pdf]

Table S2A: Values for quantitation of percent ciliated CK5+ and CK5- epithelial/cancer cells in normal, PIN, cancer, and perineural.

**Boxplot CK5+ epithelial/cancer cells**						
	n (patients)	% cilia median	n (total nuclei)	Range nuclei/patient	Range cilia/patient	P-value
Normal	10	28.8	1624	58-418	20-85	n/a
PIN	24	25.2	2968	37-406	6-102	0.14
PIN LG	13	25.6	1448	20-319	12-100	n/a
PIN HG	18	25.6	1538	36-297	6-75	n/a
Ca	35	8.1	853	1-160	0-40	**<0.0001**
Ca LG	13	0	161	1-39	0-11	n/a
Ca HG	22	11.8	692	1-160	0-40	n/a
Peri	6	0	104	1-55	0	**<0.0001**
Average	18	15.6	1174	19-232	6-65	trend**<0.0001**
**Boxplot CK5- epithelial/cancer cells**						
Normal	10	2.9	4893	270-726	3-33	n/a
PIN	24	2.5	16438	250-1527	0-70	0.4
PIN LG	13	2.7	6864	250-1386	5-62	n/a
PIN HG	18	2.0	9574	229-1462	0-30	n/a
Ca	75	1.7	52024	112-1874	0-59	**0.02**
Ca LG	35	1.9	18812	207-1273	1-49	n/a
Ca HG	40	1.3	33212	112-1874	0-59	n/a
Peri	18	1.7	7978	120-1338	0-72	0.116
Average	29	2.1	18724	194-1433	1-54	trend=**0.049**

Table S2B: Values for analysis of percent ciliated CK5+ and CK5- epithelial/cancer cells in normal, PIN, cancer, and perineural.

**Bar graph CK5+ epithelial/cancer cells**				
	Q1 n(patients)	Q1 % (patients)	Q4 n (patients)	Q4 % (patients)
Normal	2	20	2	20
PIN	15	62.5	3	12.5
PIN LG	7	53.9	2	15.4
PIN HG	11	61.1	3	16.7
Ca	29	82.9	2	5.7
Ca LG	11	84.6	1	7.7
Ca HG	18	81.8	1	4.5
Peri	6	100	0	0
Q1 ≤26.9%, Q4>33.6%				
**Bar graph CK5- epithelial/cancer cells**				
Normal	2	20	2	20
PIN	8	33.3	5	20.8
PIN LG	3	23.1	2	15.3
PIN HG	7	38.9	4	22.2
Ca	36	48	7	9.3
Ca LG	15	42.9	3	8.6
Ca HG	21	52.5	4	10
Peri	8	44.4	3	16.7
Q1 ≤1.5%, Q4>4.2%				
