# Supplementary material for: Primary Cilia Are Lost in Preinvasive and Invasive Prostate Cancer
Source: PLoS One. 2013 Jul 2;8(7):e68521. doi: 10.1371/journal.pone.0068521 (PMC3699526; doi:10.1371/journal.pone.0068521)
Supplement: Table S3 — The data in this table corresponds to Figure 3 (Table S3A corresponds to Figure 3C boxplots, Table S3B corresponds to Figure 3C bar graphs). Figure 3C depicts boxplots of the percent of ciliated stromal cells per patient for each tissue type: normal, prostatic intraepithelial neoplasia (PIN), cancer (Ca), and perinerual invasion (Peri). Bar graphs in Figure 3C depict the percent of patients with an abnormally high percent cilia (greater than the 75th percentile for normal tissue ; Q4) or an abnormally low percent cilia (less than or equal to the 25th percentile for normal tissue; Q1). Statistical analyses were not performed for LG and HG PIN and cancer separated, so no p-value was obtained for the individual grades. (PDF) [file pone.0068521.s009.pdf]

Table S3A: Values for quantitation of percent ciliated stromal cells in normal, PIN, cancer, and perineural.

**Boxplot Stromal cells**						
	n (patients)	% cilia median	n (total nuclei)	Range nuclei/patient	Range cilia/patient	P-value
Normal	10	5.9	2625	170-424	3-52	n/a
PIN	24	5.8	6332	49-509	1-46	0.71
PIN LG	13	7.9	2686	26-509	0-46	n/a
PIN HG	18	4.4	3646	46-458	1-36	n/a
Ca	75	5.2	24177	28-918	0-76	0.21
Ca LG	35	6.2	8175	51-761	0-76	n/a
Ca HG	40	4.5	16002	28-918	0-70	n/a
Peri	18	5.0	5555	136-809	0-45	0.6
Average	29	5.6	8650	67-663	1-56	trend=0.32

Table S3B: Values for analysis of percent ciliated stromal cells in normal, PIN, cancer and perineural.

**Bar graph Stromal cells**				
	Q1 n(patients)	Q1 % (patients)	Q4 n (patients)	Q4 % (patients)
Normal	2	20	2	20
PIN	9	37.5	4	16.7
PIN LG	3	23.1	4	30.8
PIN HG	8	44.4	2	11.1
Ca	32	42.7	13	17.3
Ca LG	13	37.1	7	20
Ca HG	19	47.5	6	15
Peri	8	44.4	6	33.3
Q1 ≤4.2%, Q4 >8.9%				
