# Supplementary material for: Primary Cilia Are Lost in Preinvasive and Invasive Prostate Cancer
Source: PLoS One. 2013 Jul 2;8(7):e68521. doi: 10.1371/journal.pone.0068521 (PMC3699526; doi:10.1371/journal.pone.0068521)
Supplement: Table S4 — The data in this table corresponds to Figure 4 (Table S4A corresponds to Figure 4C,D,E,F boxplots, Table S4B corresponds to Figure 4C,D,E,F bar graphs). Figure 4C,D,E,F depicts boxplots of median cilia lengths per patient broken up into cells type (all epithelial/cancer, CK5+, CK5-, stromal) for each tissue type: normal, prostatic intraepithelial neoplasia (PIN), cancer (Ca), and perinerual invasion (Peri). Bar graphs in Figure 4C,D,E,F depict the percent of patients with abnormally long cilia (greater than the 75th percentile for normal tissue ; Q4) or abnormally short cilia (less than or equal to the 25th percentile for normal tissue; Q1). (PDF) [file pone.0068521.s010.pdf]

Table S4A: Values for quantitation of cilia lengths in normal, PIN, cancer and perineural.

**Boxplot all epithelial/cancer cells**						
	n (patients with cilia)	n (total patients)	n (total cilia)	Median median cilia length (µm)	Range median cilia length (µm)	P-value
Normal	10	10	592	1.30	1.19-1.78	n/a
PIN	24	24	1385	1.22	0.73-1.63	0.15
PIN LG	13	13	679	1.32	0.75-1.63	0.74[a]
PIN HG	18	18	706	1.21	0.73-1.55	**0.005**[a]
Ca	74	75	1166	1.0	0.34-2.09	**<0.0001**
Ca LG	35	35	431	0.86	0.34-2.09	**<0.0001**[a]
Ca HG	39	40	735	1.06	0.58-1.7	0.15[a]
Peri	14	18	247	0.76	0.46-1.74	**<0.0001**
Average	29	29	730	1.09	0.64-1.78	trend**<0.0001**
**Boxplot CK5+ cells**						
Normal	10	10	445	1.38	0.77-1.75	n/a
PIN	24	24	911	1.38	0.51-2.27	0.615
PIN LG	9	13	395	1.36	1.02-1.67	0.89[a]
PIN HG	15	18	377	1.39	0.51-2.27	0.41[a]
Ca	22	75	154	1.31	0.59-4.21	0.77
Ca LG	5	35	25	0.93	0.85-1.34	**<0.0001**[a]
Ca HG	17	40	129	1.36	0.59-4.21	0.34[a]
Peri	0	18	0	n/a	n/a	n/a
Average	11	29	287	1.3	0.69-2.53	trend=0.998
**Boxplot CK5- cells**						
Normal	10	10	147	1.2	0.69-2.1	n/a
PIN	24	24	474	0.85	0.42-1.73	**0.049**
PIN LG	9	13	211	1.04	0.71-1.54	0.99[a]
PIN HG	15	18	191	0.73	0.42-1.73	**<0.0001**[a]
Ca	74	75	1012	0.93	0.34-2.09	0.093
Ca LG	35	35	406	0.82	0.34-2.09	**<0.0001**[a]
Ca HG	39	40	606	1.01	0.58-1.7	0.997[a]
Peri	14	18	247	0.76	0.40-1.74	**0.001**
Average	28	29	403	0.92	0.49-1.84	trend=**0.047**

**Boxplot stromal cells**						
	n (patients with cilia)	n (total patients)	n (total cilia)	Median median cilia length (µm)	Range median cilia length (µm)	P-value
Normal	10	10	178	1.16	0.71-1.66	n/a
PIN	24	24	377	0.93	0.51-1.79	0.41
PIN LG	9	13	196	1.04	0.81-1.79	0.85[a]
PIN HG	15	18	181	0.89	0.51-1.58	0.16[a]
Ca	70	75	1316	0.99	0.33-1.73	0.72
Ca LG	33	35	506	1.01	0.33-1.69	0.79[a]
Ca HG	37	40	810	0.98	0.68-1.73	0.69[a]
Peri	17	18	343	0.86	0.47-1.53	0.25
Average	27	29	487	0.98	0.54-1.69	trend=0.43

a- A separate linear regression analysis was performed on data for separated grades of PIN and Ca

Table S4B: Values for analysis of cilia lengths in normal, PIN, cancer and perineural.

**Bar graph all epithelial/cancer cells**				
	Q1 n (patients)	Q1 % (patients)	Q4 n (patients)	Q4 % (patients)
Normal	2	20	2	20
PIN	15	48.4	0	0
PIN LG	5	38.5	0	0
PIN HG	10	55.6	0	0
Ca	52	70.3	2	2.7
Ca LG	29	82.9	1	2.9
Ca HG	23	59.0	1	2.6
Peri	11	78.6	1	7.1
Q1 ≤1.23 µm, Q4>1.7 µm				
**Bar graph CK5+ cells**				
Normal	2	20	2	20
PIN	9	37.5	8	33.3
PIN LG	3	33.3	3	33.3
PIN HG	6	40	5	33.3
Ca	12	54.5	8	36.4
Ca LG	4	80	0	0
Ca HG	8	47.1	8	47.1
Peri	n/a	n/a	n/a	n/a
Q1 ≤1.32 µm, Q4>1.5 µm				
**Bar graph CK5- cells**				
Normal	2	20	2	20
PIN	12	50	2	8.3
PIN LG	2	22.2	1	11.1
PIN HG	10	66.7	1	6.7
Ca	30	40.5	13	17.6
Ca LG	19	54.3	5	14.3
Ca HG	11	28.2	8	20.5
Peri	9	64.3	2	14.3
Q1 ≤0.87 µm, Q4 >1.4 µm				
**Bar graph stromal cells**				
	Q1 n (patients)	Q1 % (patients)	Q4 n (patients)	Q4 % (patients)
Normal	2	20	2	20
PIN	15	62.5	2	8.3
PIN LG	4	44.4	1	11.1
PIN HG	11	73.3	1	6.7
Ca	43	61.4	4	5.7
Ca LG	18	54.5	1	3.0
Ca HG	25	67.6	3	8.1
Peri	5	83.3	0	0
Q1 ≤1.04 µm, Q4 >1.54 µm				
