# Supplementary material for: Primary Cilia Are Lost in Preinvasive and Invasive Prostate Cancer
Source: PLoS One. 2013 Jul 2;8(7):e68521. doi: 10.1371/journal.pone.0068521 (PMC3699526; doi:10.1371/journal.pone.0068521)
Supplement: Table S5 — The data in this table corresponds to Figure S5 (Table S5A corresponds to Figure S5A,B,C,D boxplots, Table S5B corresponds to Figure S5A,B,C,D bar graphs). Figure S5 depicts boxplots of the percent of ciliated cells per cell type (all epithelial/cancer, CK5+, CK5-, stromal) per patient for each tissue type: normal tissue, normal tissue adjacent to cancer (N Adj. Ca), and benign prostatic hyperplasia (BPH). Bar graphs in Figure S5A,B,C,D depict the percent of patients with an abnormally high percent cilia (greater than the 75th percentile for normal tissue ; Q4) or an abnormally low percent cilia (less than or equal to the 25th percentile for normal tissue; Q1). (PDF) [file pone.0068521.s011.pdf]

Table S5A: Values for quantitation of percent ciliated epithelial/cancer and stromal cells in normal, normal adjacent to cancer, and BPH.

**Boxplot epithelial/cancer cells**						
	n (patients)	% cilia median	n (total nuclei)	Range nuclei/patient	Range cilia/patient	P-value
Normal	10	8.9	6517	405-1097	25-95	n/a
Normal adj. ca	16	11.1	7491	92-1085	9-136	0.257
BPH	8	10.4	5750	180-1833	9-156	0.231
Average	11	10.1	6586	67-1338	14-129	
**Boxplot CK5+ cells**						
Normal	10	28.8	1624	58-418	20-85	n/a
Normal adj. ca	16	31.3	2033	29-382	7-118	0.858
BPH	8	27.1	1175	14-380	5-55	0.349
Average	11	29.1	1611	34-393	11-86	
**Boxplot CK5- cells**						
Normal	10	2.9	4893	270-726	3-33	n/a
Normal adj. ca	16	3.5	5458	63-703	2-44	0.130
BPH	8	6.1	4575	166-1453	4-102	0.08
Average	11	4.1	4975	166-961	3-60	
**Boxplot Stromal cells**						
Normal	10	5.9	2625	170-424	3-52	n/a
Normal adj. ca	16	8.0	3206	67-459	6-36	0.152
BPH	8	4.3	1716	65-427	1-23	0.125
Average	11	6.1	2516	101-437	3-37	

Table S5B: Values for analysis of percent ciliated epithelial/cancer and stromal cells in normal, normal adjacent to cancer, and BPH.

**Bar graph epithelial/cancer cells**				
	Q1 n(patients)	Q1 % (patients)	Q4 n (patients)	Q4 % (patients)
Normal	2	20	2	20
Normal adj. ca	3	18.8	7	43.8
BPH	3	37.5	4	50
Q1 ≤7.6%, Q4>11.4%				
**Bar graph CK5+ cells**				
Normal	2	20	2	20
Normal adj. ca	6	37.5	6	37.5
BPH	4	50	2	25
Q1 ≤26.9%, Q4>33.6%				
**Bar graph CK5- cells**				
Normal	2	20	2	20
Normal adj. ca	1	6.3	7	43.8
BPH	1	12.5	5	62.5
Q1 ≤1.5%, Q4>4.2%				
**Bar graph stromal cells**				
Normal	2	20	2	20
Normal adj. ca	2	12.5	7	43.8
BPH	4	50	0	0
Q1 ≤4.2%, Q4>8.9%				
