# Supplementary material for: Primary Cilia Are Lost in Preinvasive and Invasive Prostate Cancer
Source: PLoS One. 2013 Jul 2;8(7):e68521. doi: 10.1371/journal.pone.0068521 (PMC3699526; doi:10.1371/journal.pone.0068521)
Supplement: Table S6 — The data in this table corresponds to Figure S6 (Table S6A corresponds to Figure S6A,B,C,D boxplots, Table S6B corresponds to Figure S6A,B,C,D bar graphs). Figure S6 depicts boxplots of median cilia lengths per cell type (all epithelial/cancer, CK5+, CK5-, stromal) per patient for each tissue type: normal tissue, normal tissue adjacent to cancer (N Adj. Ca), and benign prostatic hyperplasia (BPH). Bar graphs in Figure S6A,B,C,D depict the percent of patients with abnormally long cilia (greater than the 75th percentile for normal tissue ; Q4) or abnormally short cilia (less than or equal to the 25th percentile for normal tissue; Q1). (PDF) [file pone.0068521.s012.pdf]

Table S6A: Values for quantitation of cilia lengths in normal, normal adjacent to cancer, and BPH.

**Box plot epithelial/cancer cells**						
	n (patients with cilia)	n (total patients)	n (total cilia)	Median median cilia length (µm)	Range median cilia length (µm)	P-value
Normal	10	10	592	1.30	1.19-1.78	n/a
Normal adj. ca	16	16	860	1.32	0.49-2.17	0.86
BPH	8	8	546	1.24	0.32-1.92	**0.048**
Average	11	11	666	1.29	0.67-1.96	
**Boxplot CK5+ cells**						
Normal	10	10	445	1.38	0.77-1.75	n/a
Normal adj. ca	16	16	620	1.33	0.79-1.71	0.975
BPH	8	8	265	1.08	0.39-1.92	0.274
Average	11	11	443	1.26	0.65-1.79	
**Boxplot CK5- cells**						
Normal	10	10	147	1.2	0.69-2.1	n/a
Normal adj. ca	16	16	240	1.08	0.49-2.17	0.79
BPH	8	8	281	1.0	0.32-1.2	0.29
Average	11	11	223	1.09	0.5-1.82	
**Boxplot Stromal cells**						
Normal	10	10	178	1.16	0.71-1.66	n/a
Normal adj. ca	16	16	249	0.97	0.74-1.92	0.596
BPH	8	8	64	0.98	0.36-1.46	0.779
Average	11	11	164	1.04	0.6-1.68	

Table S6B: Values for analysis of cilia lengths in normal, normal adjacent to cancer, and BPH.

**Bar graph epithelial/cancer cells**				
	Q1 n (patients)	Q1 % (patients)	Q4 n (patients)	Q4 % (patients)
Normal	2	20	2	20
Normal adj. ca	5	31.3	1	6.3
BPH	4	50	0	0
Q1 ≤1.23µm, Q4 >1.7µm				
**Bar graph CK5+ cells**				
Normal	2	20	2	20
Normal adj. ca	8	50	4	25
BPH	6	75	1	12.5
Q1 ≤1.32 µm, Q4>1.5 µm				
**Bar graph CK5- cells**				
Normal	2	20	2	20
Normal adj. ca	5	31.3	3	18.8
BPH	2	25	0	0
Q1 ≤0.87 µm, Q4 >1.4 µm				
**Bar graph stromal cells**				
Normal	2	20	2	20
Normal adj. ca	11	68.8	2	12.5
BPH	6	75	0	0
Q1 ≤1.04 µm, Q4 >1.54 µm				
