# Supplementary material for: Primary Cilia Are Lost in Preinvasive and Invasive Prostate Cancer
Source: PLoS One. 2013 Jul 2;8(7):e68521. doi: 10.1371/journal.pone.0068521 (PMC3699526; doi:10.1371/journal.pone.0068521)
Supplement: Table S7 — Nuclear β-catenin was correlated to capsular penetration and biochemical recurrence using a two-by-two table. Biochemical recurrence was defined as free serum PSA >0.1 ng/ml for two consecutive measurements. ≤75th percentile of nuclear β -catenin of normal basal cells is considered low/moderate nuclear β -catenin, while >75th percentile of nuclear β -catenin of normal basal cells is considered high nuclear β -catenin. (PDF) [file pone.0068521.s013.pdf]

Table S7: Statistically significant correlation between patient characteristics and high or moderate/low nuclear β-catenin in cancers.

	≤75th percentile of nuclear β -catenin of normal basal cells	>75th percentile of nuclear β -catenin of normal basal cells	P-value
**% patients with capsular penetration**	2.6	40	**<0.0001**
**% patients with biochemical recurrence**	68.4	20	**0.001**
**Total n (patients)**	38	15	
