# Supplementary material for: Primary Cilia Are Lost in Preinvasive and Invasive Prostate Cancer
Source: PLoS One. 2013 Jul 2;8(7):e68521. doi: 10.1371/journal.pone.0068521 (PMC3699526; doi:10.1371/journal.pone.0068521)
Supplement: Table S8 — Patient characteristics were correlated to percent ciliated epithelial cells in normal tissue adjacent to cancer using linear regression. Number of patients =16. (PDF) [file pone.0068521.s014.pdf]

Table S8: Correlation between patient characteristics and percent cilia in all epithelial cells in normal adjacent to cancer.

Patient Characteristics	P-value	β	95% Confidence Interval
Age	0.604	-0.005	(-0.028, 0.017)
Tumor stage	0.834	0.028	(-0.257,0.314)
Capsular penetration	0.989	-0.002	(-0.396, 0.391)
Biochemical recurrence	0.671	0.069	(-0.271, 0.409)
Months to biochemical recurrence	Insufficient data Regression model does not fit	-	-
Tumor size of largest tumor	0.209	-0.004	(-0.011, 0.003)
Pre-operative free PSA	0.087	-0.036	(-0.079,0.006)
