# Supplementary material for: Primary Cilia Are Lost in Preinvasive and Invasive Prostate Cancer
Source: PLoS One. 2013 Jul 2;8(7):e68521. doi: 10.1371/journal.pone.0068521 (PMC3699526; doi:10.1371/journal.pone.0068521)
Supplement: Table S9 — Patient characteristics were correlated to percent ciliated CK5+ epithelial cells in normal tissue adjacent to cancer using linear regression. Number of patients =16. (PDF) [file pone.0068521.s015.pdf]

Table S9: Correlation between patient characteristics and percent cilia in CK5+ epithelial cells in normal adjacent to cancer.

Patient Characteristics	P-value	β	95% Confidence Interval
Age	0.725	0.004	(-0.022,0.031)
Tumor stage	**0.037**	0.22	(0.016,0.437)
Capsular penetration	0.134	0.25	(-0.085, 0.582)
Biochemical recurrence	**0.04**	0.26	(0.013, 0.501)
Months to biochemical recurrence	Insufficient data Regression model does not fit	-	-
Tumor size of largest tumor	0.309	0.004	(-0.004, 0.013)
Pre-operative free PSA	0.747	0.010	(-0.055,0.075)
