# Supplementary material for: Primary Cilia Are Lost in Preinvasive and Invasive Prostate Cancer
Source: PLoS One. 2013 Jul 2;8(7):e68521. doi: 10.1371/journal.pone.0068521 (PMC3699526; doi:10.1371/journal.pone.0068521)
Supplement: Table S10 — Patient characteristics were correlated to percent ciliated CK5-epithelial cells in normal tissue adjacent to cancer using linear regression. Number of patients =16. (PDF) [file pone.0068521.s016.pdf]

Table S10: Correlation between patient characteristics and percent cilia in CK5- epithelial cells in normal adjacent to cancer.

Patient Characteristics	P-value	β	95% Confidence Interval
Age	0.369	-0.016	(-0.022,0.031)
Tumor stage	0.432	-0.17	(0.616,0.277)
Capsular penetration	0.369	-0.25	(-0.837, 0.329)
Biochemical recurrence	0.661	-0.012	(-0.683, 0.446)
Months to biochemical recurrence	Insufficient data Regression model does not fit	-	-
Tumor size of largest tumor	**0.015**	-0.013	(-0.022, -0.027)
Pre-operative free PSA	**0.002**	-0.083	(-0.130,-0.035
