# Supplementary material for: Primary Cilia Are Lost in Preinvasive and Invasive Prostate Cancer
Source: PLoS One. 2013 Jul 2;8(7):e68521. doi: 10.1371/journal.pone.0068521 (PMC3699526; doi:10.1371/journal.pone.0068521)
Supplement: Table S11 — Patient characteristics were correlated to percent ciliated CK5+ epithelial cells in PIN using linear regression. Number of patients =24. (PDF) [file pone.0068521.s017.pdf]

Table S11: Correlation between patient characteristics and percent cilia in CK5+ PIN cells.

Patient Characteristics	P-value	β	95% Confidence Interval
Age	0.332	0.008	(-0.009, 0.025)
Tumor stage	0.06	-0.21	(-0.423,0.006)
Capsular penetration	0.06	-0.24	(-0.500, 0.008)
Biochemical recurrence	0.10	-0.51	(-1.12, 0.109)
Months to biochemical recurrence	Insufficient data Regression model does not fit	-	-
Tumor size of largest tumor	**0.006**	-0.016	(-0.027, -0.005)
Pre-operative free PSA	0.552	-0.012	(-0.055,0.030
