# Supplementary material for: Primary Cilia Are Lost in Preinvasive and Invasive Prostate Cancer
Source: PLoS One. 2013 Jul 2;8(7):e68521. doi: 10.1371/journal.pone.0068521 (PMC3699526; doi:10.1371/journal.pone.0068521)
Supplement: Table S12 — Patient characteristics were correlated to percent ciliated cancer cells using linear regression. Number of patients =75. (PDF) [file pone.0068521.s018.pdf]

Table S12: Correlation between patient characteristics and percent cilia in all cancer cells in prostate cancers.

Patient Characteristics	P-value	β	95% Confidence Interval
Age	0.943	0.001	(-0.028, 0.030)
Tumor stage	0.651	-0.097	(-0.527,0.331)
Capsular penetration	0.816	-0.068	(-0.652, 0.515)
Biochemical recurrence	0.932	0.018	(-0.404, 0.440)
Months to biochemical recurrence	0.958	-0.0001	(–0.006,0.006)
Tumor size of largest tumor	0.317	-0.013	(-0.038,0.013)
Pre-operative free PSA	0.333	-0.003	(-0.011,0.003)
